# Advanced Bionic 3D Interfacial Solar Steam Generator With One‐way Water Supply for Highly Efficient Desalination and Oil‐Fouling Prevention

**DOI:** 10.1002/advs.202412545

**Published:** 2024-12-25

**Authors:** Yiming Bu, Xin Li, Weiwei Lei, Hongjun Yang, Weilin Xu, Qi Han, Jingliang Li

**Affiliations:** ^1^ Institute for Frontier Materials Deakin University Geelong VIC 3216 Australia; ^2^ Key Laboratory of Green Processing and Functional New Textile Materials of Ministry of Education Wuhan Textile University Wuhan 430200 China; ^3^ School of Science RMIT University City Campus Melbourne VIC 3000 Australia

**Keywords:** anti‐oil‐fouling, biomimetic structure, interfacial solar steam generation, seawater desalination

## Abstract

Interfacial solar steam generation (ISSG) employed for seawater desalination and wastewater purification shows great promise to alleviate global freshwater scarcity. However, simultaneous optimization of water transfer direction in a cost‐effective and reliable ISSG to balance thermal localization, salt accumulation, and resistance to oilfouling represents a rare feat. Herein, inspired by seabird beaks for unidirectional water transfer, eco‐friendly and cost‐effective plant extracts, sodium alginate, and tannic acid, are selected for crafting an innovative Sodium Alginate‐Tannic Acid Hemispheric Evaporator (STHE). The STHE aligned with centripetally tapered channels ensures one‐directional water flow and effectively inhibits downward heat transfer, thereby boosting energy efficiency. Additionally, the integration of one‐way water supply in tapered channels with interfacial evaporation of STHE, mimicking plant transpiration, collaboratively facilitates upward water transfer for a reliable solar‐driven water evaporation rate of ≈2.26 kg m^−2^ h^−1^ under one sun irradiation. Even in a brine of 15.0 wt % solution, no salt crystals are observed on the surface of STHE. Hemispheric structure and superhydrophilicity are conducive to oil repellence. This work provides pivotal inspiration for constructing next‐generation solar generators of high‐efficiency, salt‐tolerance, and anti‐oil‐fouling.

## Graphical abstract

Inspired by shorebird beaks and tree transpiration, this 3D sodium alginate‐tannic acid hemispherical evaporator with a one‐way water supply boosts interfacial solar steam generation, reduces water back diffusion, and minimizes heat loss. This design uniquely combines biomimetic water transport, enhanced osmotic pressure, and thermal management in a cohesive system that is optimized for long‐term use in high‐salinity and oily environments.

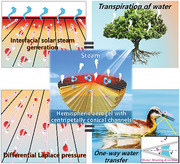

## 1 Introduction

The rising global demand for freshwater, exacerbated by climate change and limited access to affordable purification systems, has made water scarcity a critical global challenge.^[^ ^1^, ^2^ ^]^ For decades, traditional thermal distillation and membrane‐based reverse osmosis technologies have played vital roles in water treatment. However, their practical application is limited by high energy consumption, complex treatment processes, significant costs, and the risk of secondary pollution from fuel combustion or waste membranes, especially in developing countries and regions.^[^ ^3^, ^4^, ^5^, ^6^ ^]^

Interfacial solar steam generation (ISSG) as an emerging effective strategy for freshwater production has been developed to alleviate worldwide water scarcity owing to its minimal carbon footprint and higher efficiency by localization of heat on the air‐water interface.^[^ ^7^, ^8^, ^9^, ^10^ ^]^ The past few years have seen a dramatic advance in boosting the water evaporation rate and light‐to‐heat conversion efficiency of ISSG by selection of photothermal materials for improving light absorption,^[^ ^11^, ^12^, ^13^ ^]^ minimization of heat loss,^[^ ^14^, ^15^, ^16^ ^]^ optimizing water supply,^[^ ^17^, ^18^ ^]^ reducing evaporation enthalpy and constructing cold evaporation surfaces.^[^ ^10^, ^19^, ^20^, ^21^ ^]^ Yet, existing ISSG systems still struggle to maintain efficiency and functionality when exposed to complex pollutants like salts and oil.^[^ ^22^, ^23^ ^]^ Hence, the development of effective ISSG devices that ensure high efficiency, stability, and reliability remains a priority.

To address salt resistance, ISSG systems have been advanced by “salting‐out” strategies, including physical cleaning,^[^ ^24^, ^25^ ^]^ periodic operation,^[^ ^26^ ^]^ local crystallization,^[^ ^27^ ^]^ and gravity‐assisted cleaning,^[^ ^28^ ^]^ which are limited by high production costs and excessive dependence on passive diffusion. Alternatively, “salt‐free” methods, such as ion rejection and non‐contact ISSG devices, offer salt tolerance but typically suffer from low evaporation rates.^[^ ^29^, ^30^ ^]^ Hydrophobic surface modification of evaporators^[^ ^31^ ^]^ and the adoption of back diffusion and convection,^[^ ^32^ ^]^ are currently standout methods for achieving effective salt resistance in ISSG systems. Hydrophobic designs, while effective at concealing salts, become less efficient with high‐salinity water over time, as salt buildup impedes water transport and reduces evaporation efficiency.^[^ ^33^, ^34^ ^]^ Notably, back diffusion and convection facilitate the return of salt to the bulk water but also cause heat loss, reducing system efficiency.^[^ ^35^ ^]^ Thus, effective regulation of water transport is crucial for the timely replenishment of salty water on the surface of an ISSG, requiring a sufficient water supply. The ISSG systems with tortuous or randomly aligned water pathways often lead to insufficient water flow due to higher water transfer resistance, thereby promoting salt precipitation.^[^ ^36^ ^]^ Furthermore, ISSG devices with plentiful vertically aligned water pathways, intended for back diffusion and convection, may suffer from excessive water supply. This can lead to significant conduction heat loss to the bottom bulk water, ultimately resulting in a reduction of evaporation rates.^[^ ^37^ ^]^ Therefore, crafting ISSG devices with flexible pathways to optimize water supply and minimize downward heat loss remains a key challenge.

**Scheme 1:**
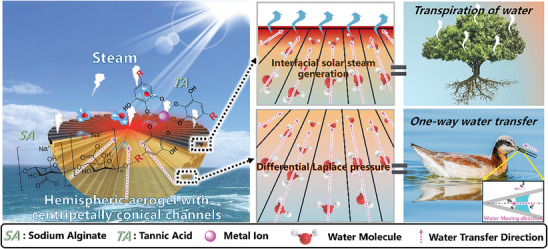
Biomimetic design of STHE showcasing the detailed mechanism of water transportation in tapered channels.

Herein, inspired by the beaks of shorebirds for one‐way water transfer, a 3D free‐standing Sodium Alginate‐Tannic Acid Hemispheric Evaporator (STHE) has been engineered with spatially distributed tapered channels for directional water transfer by a multidirectional freeze‐casting (MDF) approach to achieve highly efficient ISSG. The spatially centripetal alignments in STHE ensure one‐directional water transfer, effectively inhibiting downward heat transfer and thereby enhancing energy efficiency (**Scheme** **1**). The size of the well‐aligned channels can be adjusted by varying the concentration of sodium alginate (SA), enabling the attainment of a balance between water supply and evaporation rate. To enable reliable solar‐driven water evaporation, tannic acid (TA) was opted for chelation with Fe^3+^ (MPN) to form a photothermal layer due to its stability, durability, and efficiency, as well as environmentally friendly, cost‐effective, and multifunctional nature. This choice offers enhanced performance in desalination and oil‐fouling prevention compared to traditional carbon‐based materials. Thus, the interfacial evaporation of STHE also drives upward water transportation mimicking the transpiration effects of plants, which ensures a high evaporation rate of 2.26 kg m^−2^ h^−1^ under one sun irradiation. The STHE exhibited stable performance after one month in seawater, with no structural degradation and a steady evaporation rate of 2.10 kg m^−2^ h^−1^, indicating its long‐term reliability in harsh environments. Because of the rich anions and cations in the ion‐crosslinked SA‐TA networks, STHE exhibits excellent salt repelling properties even in a brine of 15.0 wt % solution. The underwater anti‐oil‐fouling function is achieved through the superoleophobicity of the SA‐TA network, which is based on hydrophilic micro‐nano structure‐induced Cassie contact. The intricately constructed hemisphere also contributes significantly to preventing oil‐fouling, facilitating the easy roll‐off of underwater oil droplets along the curved surface of the STHE hemisphere. Thus, this design holds significant value in promoting solar‐driven clean water generation technology and addressing contaminated hydrological environments.

## 2 Results and Discussion

### 2.1 Preparation of SA‐TA Hemisphere (STH)

**Figure 1:**
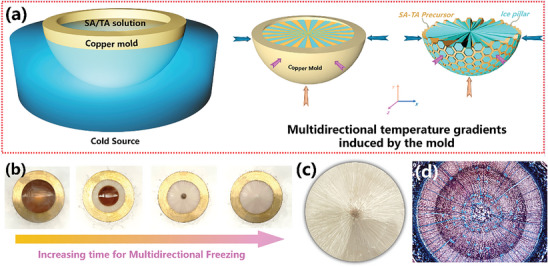
The fabrication of STH. a) Schematic illustration of the synthetic route for the STH (The hexagonal shape in **Figure** **1**a was chosen as a conceptual representation of ice pillar and does not hold specific functional significance in this design); b) Freezing process with a multidirectional freeze‐casting strategy; c) Digital image of the dry, as‐prepared STH, showing its precise and well‐defined structural alignments; d) Optical microscope image of the cross‐section of Pinus steam (pine tree) (Nikon, 2003 Photomicrography Competition, Licensed under Nikon, https://www.nikonsmallworld.com/galleries/2003‐photomicrography‐competition/cross‐section‐of‐pinus‐stem‐pine‐tree).

The free‐standing hemispheric SA‐TA matrix with spatially centripetal alignments of tapered channels was first prepared by directional ice crystal growth and freeze drying, shown in **Figure** **1**a,b. A cylindrical copper rod with a 3.0 cm diameter hemispheric depression filled with SA‐TA aqueous dispersion was immersed in liquid nitrogen to enable fast freezing across the entire curve of the hemispheric depression. During this period, multidirectional temperature gradients (*x, y, z*) along the radius of the hemisphere play a crucial role in shaping the centripetal alignments of tapered channels, closely resembling the intricate patterns of the Pinus steam (Figure 1c,d). Ice crystals typically grow in a lamellar style along specific crystallographic directions, which coincide with the temperature gradient direction.^[^ ^38^ ^]^ In this case, ice pillar nucleation commenced at the outer surface of the hemisphere, leading to the growth of ice crystals in a spatially centripetal and radially planar direction along the temperature gradients toward the centre of the mould. During this time, the SA‐TA solution was propelled ahead slowly by ice crystals in the freezing direction in time (Figure 1b), then the inward spatially centripetal arrangements of ice crystals were replicated to form lamellar tapered channels after sublimation (Figure 1c). Experimental details are provided in Supporting Information. Note that STHE refers to the SA‐TA hemisphere after the chelation of tannic acid (TA) with Fe^3+^ serving as a photothermal conversion layer. STH refers to the SA‐TA hemisphere without a photothermal conversion layer.

### 2.2 Characterization of STH

**Figure 2:**
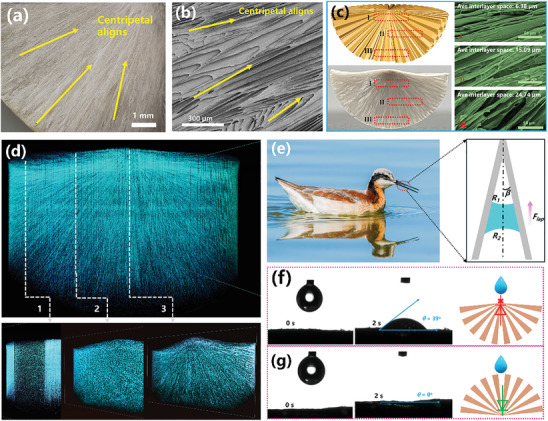
Characterization and One‐way water transfer property of STH. a) Optical microscopy image of STH; b) SEM image showing the top surface of the STH; c) SEM image showing the cross‐sectional surface of the STH; d) Micro‐CT images of the internal 3D structure of the STH; e) Digital image of the beak of a shorebird, highlighting its structural adaptation for efficient one‐way water transport (source from Lives of North American Birds by Kenn Kaufman©1996, Licence under Houghton Mifflin Harcourt Publishing Company). Water contact angle images of the STH surfaces, depicting measurements from the top‐to‐bottom orientation f) and the bottom‐to‐top orientation (g).

This STHE scaffold showed spatially centripetal alignments of tapered channels, which were characterized by scanning electron microscopy, optical microscopy, and Micro‐CT (**Figure** **2**a–d). The geometric structure of the hemispheric mould causes lamellar ice crystals to grow preferentially not only in the planar centripetal direction but also in the spatial centripetal direction. The space between the STH lamellae gradually decreases from the outer surface to the centre of the hemisphere due to spatial constraints.^[^ ^39^, ^40^ ^]^ As clearly observed in Figure 2c, the average interlayer spaces decrease from 24.74 to 6.38 µm, indicating the successful construction of radially centrosymmetric alignments with tapered structures.^[^ ^41^ ^]^ Micro‐CT, a non‐destructive 3D imaging technique, allowed for detailed visualization of the STHE scaffold's layer‐by‐layer cross‐sections (Figure 2d). The reconstructed 3D model depicts the spatial arrangement and internal structural distribution, while the 2D vertical cross‐sections reveal the porous architecture with radially aligned channels converging in a centripetal direction.

The well‐arranged tapered channels with centripetal alignments could guide water transfer in one direction to ensure a continuous water supply for highly efficient interfacial solar steam generation. The mechanism behind the one‐way water transfer in tapered shapes inspired by the beaks of shorebirds could be explained that the driving force for propelling water droplets to unidirectionally move comes from the asymmetry Laplace differential pressure in tapered channels (Figure 2e, inset: details analyse shown in Figure S1, Supporting Information).^[^ ^42^ ^]^ For further verification of the as‐prepared ISSG with tapered channels for unidirectional water transfer, water droplet diffusion on different surfaces (top to bottom and bottom to top) and also antigravity upward water transport (details shown in Figure S2, Supporting Information) were tested. As illustrated in Figure 2f, water droplets only spread out on the top surface (apex of tapered channels) instead of transferring to the bottom. It could be ascribed to the superhydrophilicity of the SA‐TA network (characterization details of the interaction of SA and TA shown in Figure S3 (Supporting Information), as well as the repelling force from the differential Laplace pressure (*F_lap_*), resulting in water droplet spreading out on the surface instead of diffusing downward. On the contrary, water droplets could be quickly absorbed on the bottom‐to‐up surface (base of tapered channels) (Figure 2g), which could be explained that the water diffusion direction is parallel to Laplace differential pressure. This is conducive to continuous water replenishment for highly efficient solar‐driven interfacial water evaporation.

The black color of the STHE arises from the iron‐phenol reaction between TA and Fe^3^⁺, forming a photothermal conversion layer through ionic crosslinking and chelation. This complexation is evidenced by significant shifts and intensity reductions in the FT‐IR spectrum, as shown in Figure S4 (Supporting Information). Specifically, the C═O stretching band of the carbonyl group shifted from 1696 to 1709 cm⁻¹, and the phenolic (O─H) deformation shifted from 1172 to 1195 cm⁻¹, indicating the TA‐Fe^3^⁺ chelation. Additionally, the C─C stretching of the benzene ring at 1609 cm⁻¹, C─C and C─O stretch at 1532 and 1446 cm⁻¹, C─O stretching of carboxylic acids/ethers at 1015 cm⁻¹, and C─H out‐of‐plane bending at 870 cm⁻¹ all showed a marked decrease in intensity.^[^ ^43^, ^44^ ^]^ These variations confirm the complexation between tannic acid and Fe^3^⁺ ions, resulting in the formation of a photothermal layer within the STHE. The ionic cross‐linking and complexation were further demonstrated by XPS.^[^ ^45^ ^]^ The wide‐scan XPS of STHE, as shown in Figure S5 (Supporting Information), reveals that carbon (C), oxygen (O), iron (Fe), and chlorine (Cl) are the predominant elements. Given that the SA‐TA matrix is gelatinized and chelated by ferric (III) chloride, the appearance of Fe 2p and Cl 2p peaks in the XPS spectrum of the STHE confirms the incorporation of ferric and chloride ions into the structure. Together with FT‐IR analysis, this result validates the successful fabrication of the photothermal STHE. Furthermore, the Fe 2p spectrum of STHE shows a Fe 2p_3/2_ peak at 713.3 eV and a Fe 2p_1/2_ peak at 725.7 eV, confirming the successful formation of the TA‐Fe^3^⁺ photothermal layer within the STHE through chemical polymerization.^[^ ^46^ ^]^

### 2.3 Property of SA‐TA Hemispheric Evaporator (STHE)

**Figure 3:**
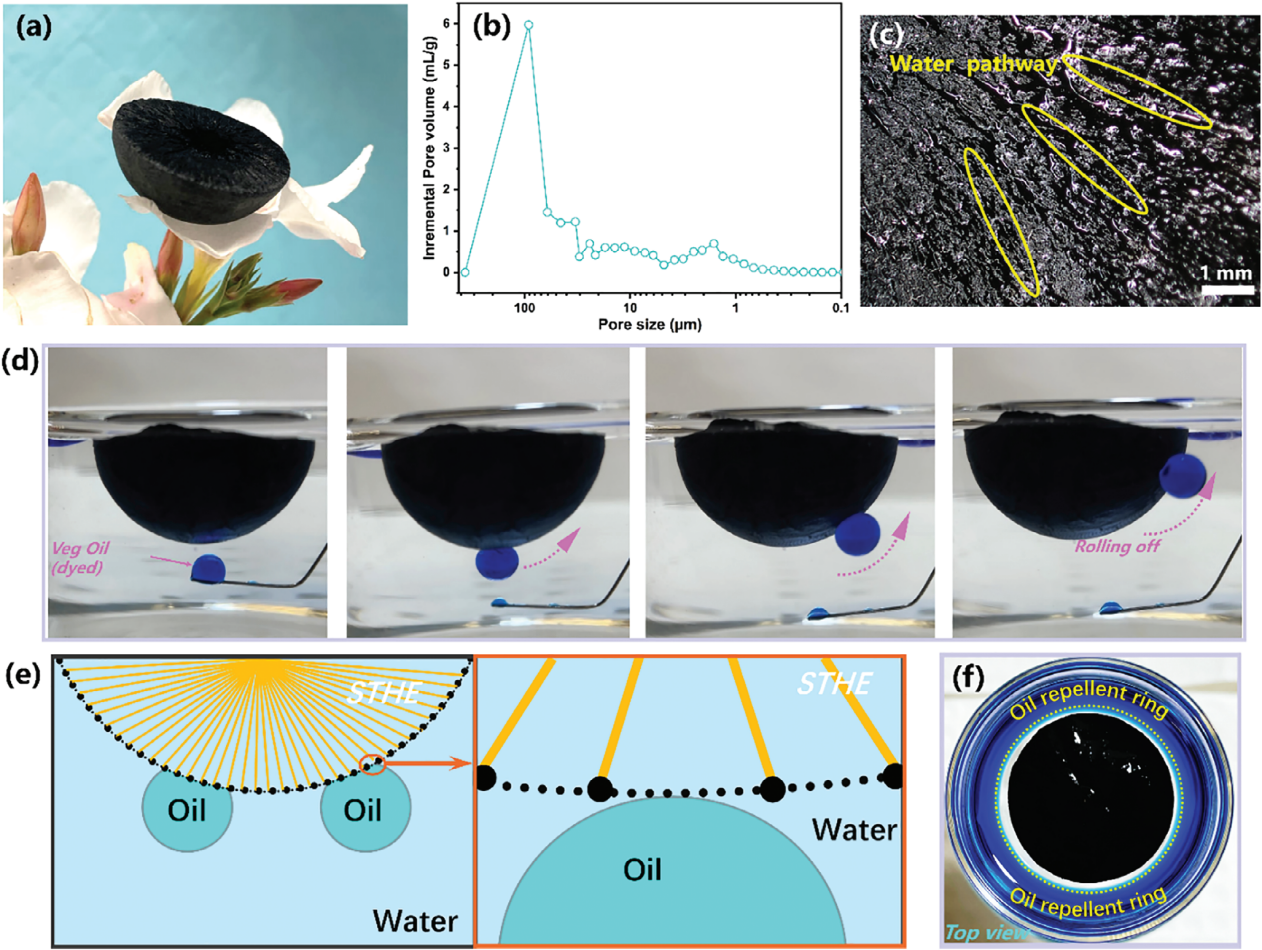
Structural and anti‐oil‐fouling properties of STHE. a) Optical image of STHE placed on the petals of a Southern magnolia; b) Pore size distribution within the STHE; c) Microscopy image of STHE; d) Images demonstrating the underwater anti‐oil‐fouling property of STHE; e) Schematic illustrating the mechanism behind the STHE's underwater anti‐oil‐fouling property; f) Image showing the oil repellent ring surrounding the STHE without fouling.

By the MDF strategy and ionic crosslinking, the free‐standing dry STHE was well prepared, where it is ultralight (with a density of 0.03 g cm^−3^) to balance on a Southern magnolia petal (**Figure** **3**a). The mercury intrusion method revealed that the as‐prepared STH scaffold had a wide distribution of pore size of 2–100 µm, alongside an average pore size of 6.45 µm and a notable porosity of 93.5% (Figure 3b). The porous structures are anticipated to facilitate steam dissipation.

Moreover, structural stability holds significant importance for applications of ISSG. Based on the compressive stress‐strain curve (Figure S6, Supporting Information), it can be observed that the STHE hydrogel is capable of withstanding 80% volume reduction and 0.2 MPa pressure, highlighting its elasticity and compliance. The supreme mechanical performance of STHE lays a solid foundation for achieving durable and reliable water evaporation and offers considerable potential for convenient storage and transportation of ISSG in practical applications. As shown in Figure S7 (Supporting Information), an STHE could be completely wetted in 10 s, where the water was transferred from the bottom to the upper surface quickly with the help of differential Laplace pressure from tapered channels. This demonstrates that the well‐built inner centripetal aligned tapered channels of STHE could ensure quick one‐way water transfer against gravity for continuous interfacial steam generation. In the optical microscope image of STHE (Figure 3c), the water pathway is clearly visible, indicating that the well‐designed centripetal alignments on the upper surface of STHE also play a vital role in guiding water transportation from the outer to the centre.

The antifouling capability of ISSG, especially against oil, is crucial for practical deployment in complex wastewater treatments. As shown in Figure 3d, dyed oil droplets predominantly maintain a spherical shape without adhering to the bottom surface of STHE. At the same time, the hemispheric shape of STHE floating on water supports anti‐fouling effects, allowing oil droplets to roll off automatically along the curved surface. This phenomenon is a result of the abundant presence of superhydrophilic groups (─COOH and ─OH) in SA‐TA, along with the micro‐nano structure formed by the sublimation of fine ice crystals (Figure S8, Supporting Information). This structure efficiently traps water within the rough surface, leading to the formation of a stable Cassie contact with oil droplets underwater (Figure 3e).^[^ ^4^ ^]^ Additionally, there is a noticeable oil‐repellent ring encircling the upper surface of STHE, providing clear evidence of its superior anti‐oil‐fouling capability (Figure 3f). That would be advantageous for the implementation of complex oily water and emulsion purification.

### 2.4 Photothermal Evaporation Performance of STHE

The effectiveness of the interfacial water evaporation process heavily relies on the photothermal conversion material and its ability to absorb light efficiently. Affordable and eco‐friendly plant extracts, such as SA from kelp and TA from tea leaves, were specifically selected to assemble the STHE. In comparison to other black nanoparticles and carbonous photothermal materials, tannic acid‐Fe^3+^ chelates, known as the category of metal‐phenolic networks (MPN), demonstrated appealing porous structures and a robust affinity to various substrates.^[^ ^47^, ^48^ ^]^ They can securely anchor onto scaffold materials and display remarkable light absorption capabilities,^[^ ^9^ ^]^ thereby showing significant potential for enhancing the stability and durability of ISSG while minimizing the risk of secondary pollution in practical applications.

**Figure 4:**
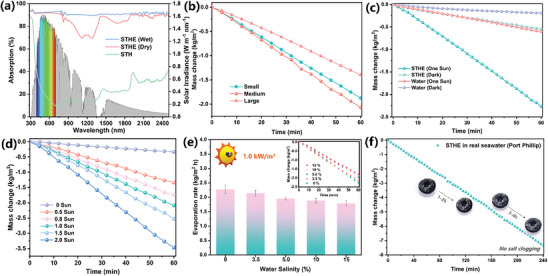
Evaporation performance of STHE. a) Solar spectrum and UV–vis–NIR diffuse reflectance patterns of STH and STHE (wet and dry); b) Water mass change over time for STHEs of different diameters under one sun illumination; c) Comparison of water mass change with and without STHE in both dark conditions and under one sun illumination; d) Water mass change over time for STHE varying sun intensities (0–2.0 sun); e) Water evaporation rates of STHE in NaCl solutions of different concentrations under one sun illumination; f) Water mass change over 4 h with STHE in real seawater under one sun illumination.

The sunlight absorption properties of the SA‐TA based hydrogels were assessed using a UV‐vis‐NIR spectrometer equipped with an integrating sphere. As shown in **Figure** **4**a, STH, without MPN as a photothermal conversion layer, exhibited only ≈40% absorbance of the standard solar spectrum (AM 1.5G). However, after incorporating MPN, the wet STHE demonstrated excellent light absorption (≈94%) across the entire spectrum, which was higher than that of a dry one. However, the surface temperature of the dry STHE rapidly rose from room temperature to 75 °C within 10 min under one sun illumination, then sharply dropped when the light was turned off (Figure S9, Supporting Information). Wet STHE exhibited relatively moderate changes in surface temperature with one sun exposure, owing to its exceptional energy utilization efficiency. This ensured the effective conversion of absorbed light into heat to facilitate water evaporation within the inner channels. This high solar light absorption property of MPN on SHT endowed the STHE with outstanding interfacial photothermal conversion properties. Under one sun (100 mW cm^−2^) illumination (Figure S9, Supporting Information), the top surface temperature of wet STHE could elevate quickly from room temperature to 32.3 °C in 10 min and kept steady at 35.2 °C in 60 min due to excellent photothermal conversion property of MPN and also the well‐built structure for thermal localization, which is conducive for efficient water evaporation. As shown in the infrared images of Figure S10 (Supporting Information), the surface temperature differences between the curved side and top of STHE drove the cold evaporation, which played a great role in further improving the interfacial solar steam evaporation rate.^[^ ^49^ ^]^ The well‐designed hemispheric ISSG showcases spatially centripetal structures with tapered channels, ensuring one‐way water transfer from the bottom water pathway to the top photothermal layer for evaporating. This prevents water refluxing, thereby minimizing heat conduction loss downward to bulk water and ensuring highly efficient water evaporation.

By tuning the diameter of the hemisphere, the STHE with different lengths of water supply channels could be accordingly manipulated. Three STHEs with different diameters [(1.5 cm (small), 3.0 cm(medium), and 4.5 cm (large)] were prepared to optimize the relationship between water supply and evaporation rate. As shown in Figure 4b and Figure S11 (Supporting Information), STHEs of different sizes exhibited varying average surface temperatures after 1 h of one sun illumination: large samples reached 29.6 °C, medium samples 35.5 °C, and small samples 31.6 °C, with their evaporation rates following the same trend. Notably, the medium‐sized STHE demonstrated the highest evaporation efficiency, indicating optimal heat utilization and conversion. In contrast, both the large and small STHE samples showed suboptimal performance, likely due to the imbalance between water supply and evaporation caused by the differing lengths of their internal channels, which affected their water transport efficiency. In the case of the medium‐sized STHE, an optimized water pathway length resulted in the highest evaporation rate compared to the other two sizes. This highlights a trade‐off between water supply flux and evaporation rate for achieving the highest energy efficiency. Therefore, unless stated otherwise, the STHE with a medium diameter was selected for further investigations in the subsequent experiments.

The solar steam generation performances of the biomimetic STHE and control samples were investigated. Figure 4c illustrates the variation in water mass changes with and without STHEs, both in darkness and under one sun irradiation. The graph clearly demonstrates a nearly linear decrease over time. Pure water experienced a mass change of 0.19 kg m^−2^ under dark conditions and 0.54 kg m^−2^ under one sun irradiation for 60 min without STHE. However, the average water evaporation rate of STHE in pure water increased greatly to 2.26 kg m^−2^ in 1 h, attributed to the supreme light‐to‐heat conversion and well‐thought‐out design of water supply systems. Compared to previous state‐of‐the‐art works, our bio‐mimetic 3D evaporator featuring centripetal alignments for one‐way water transfer, demonstrates record‐high evaporation properties (Figure S12, Supporting Information). Notably, this high level of evaporation performance is maintained even under challenging conditions, such as high salinity and oily water even salty emulsions, without salt accumulation or oil contamination over prolonged use.

To verify that the prepared centripetal structure (STHE) helps suppress heat conduction downward into the bulk water during unidirectional water transport, a similarly vertically oriented structure (ST‐V) was also fabricated and used for comparison to assess heat loss and thermal conduction (details shown in Figure S13, Supporting Information). After continuous illumination for 1h, the temperature of underlying bulk water of STHE and ST‐V elevated by 1.2 and 4.8 °C respectively, where the calculated conduction heat loss is ≈3.41% and ≈13.66% respectively. The discrepancy in heat conduction loss is mainly due to differences in longitudinal thermal conductivity arising from the distinct centripetally aligned structures. The centripetally radiant channels in the STHE evaporator significantly reduce longitudinal heat transfer, achieving a low longitudinal thermal conductivity (0.43 W m^−1^ K^−1^, hydrous), lower than that of pure water (≈0.60 W m^−1^ K^−1^). While the ST‐V enables fast water transport and backflow for dissolving salts thanks to the low tortuosity of channels, it also increases heat exchange, leading to a high longitudinal thermal conductivity (0.69 W m^−1^ K^−1^, hydrous). As a result, the low longitudinal thermal conductivity, along with the centripetal alignments that support unidirectional water transport and prevent backflow heat conduction, localizes the heat at the evaporation interface, driving rapid evaporation while minimizing heat dissipation into the bulk water below.

It's noteworthy that the natural sunlight intensity fluctuates around the theoretical value (1.0 kW m^−2^) in different locations. Therefore, it is essential to accurately evaluate the evaporation performance of STHEs with various sunlight intensities. As depicted in Figure 4d, with the increase in sunlight intensity from 0.5 to 2.0 kW m^−2^, the water mass loss in 1 h escalated from 1.73 to 3.47 kg m^−2^. This increasing trend aligns consistently with the surface temperature (Figure S14, Supporting Information). Saline water with varying concentrations of NaCl and real seawater was utilized to showcase the continuous solar‐driven salty water desalination performances of the biomimetic STHEs. The well‐designed STHE features tapered channels with low tortuosity and is aligned spatially in a centripetal manner. These channels enable one‐way upward transportation of water solely from the bottom, which endows low water transportation resistance, ensuring rapid water supplementation for continuous interfacial evaporation. Hence, as depicted in Figure 4e, the evaporation rates of STHE in saline water show a slight decrease as the NaCl concentration rose from 3.5 to 15.0 wt.%, indicating stable and reliable solar‐driven desalination. This decrease trend might be attributed to the heightened evaporation enthalpy of water as the concentration of NaCl increases.^[^ ^50^ ^]^ As shown in Figure S15, no salt crystal accumulation was observed after 1 h of solar‐driven desalination in highly saline water (15.0 wt.%). Furthermore, real seawater collected from Port Philip Bay, Victoria, Australia, was utilized to explore solar‐driven desalination of the STHE. During 4 h of exposure to one sun illumination, the STHE operating in real seawater maintained a steady water evaporation rate with a linear trend, and notably, encountered no instances of salt clogging (Figure 4f). This highlights its promising potential for continuous and practical implementation of seawater desalination. The mechanism behind the durable and reliable anti‐salt fouling could be explained by the rapid one‐way water transfer for replenishment of water evaporation. Due to stable crosslinked networks of SA‐TA‐Fe^3+^ (details shown in Figure S16, Supporting Information), these coordination bonds between SA‐TA‐Fe^3+^ remain intact without significant changes, demonstrating that no ion exchange occurs within the SA‐TA Fe^3+^ networks, even after immersing STHEs in NaCl solutions (with high concentration of 10 and 25%). Simultaneously, as depicted in Figures S17 and S18 (Supporting Information), the STHE channels oriented in the centripetal direction are densely filled with ions, owing to the abundant presence of anions (e.g., ─COO^−^) and cations (e.g., Fe^3+^) resulting from ionization within the SA‐TA‐Fe network. This results in a significant osmotic pressure (details exhibited in Figure S19, Supporting Information), facilitating the exclusive pumping of water into the STHE channels along the Laplace asymmetric force while repelling the ions in saline water,^[^ ^4^, ^51^ ^]^ avoiding salt clogging.

### 2.5 Water Activation of STHE

**Figure 5:**
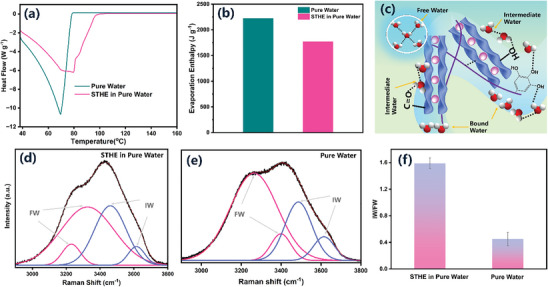
Water activation of saturated STHE in water. a) DSC curve of pure water and the saturated STHE after immersion in pure water; b) Evaporation enthalpy of pure water and the saturated STHE after immersion in pure water in pure water; c) Schematic illustration showing the water state in STHE; d) Raman spectrum displaying the fitting peaks of intermediate water (IW) and free water (FW) within STHE when immersed in pure water; e) Raman spectrum of pure water showing the fitting peaks of IW and F; f) Ratio of IW to FW in both pure water and STHE when immersed in pure water.

The phase transformation of water is influenced by the surrounding polymer networks of SA‐TA. **Figure** **5**a illustrates the differential scanning calorimetry (DSC) curves of STHE in pure water compared to bulk pure water, which serves as the control sample. It is evident that the DSC signal of pure water exhibits a sharp drop, indicating instantaneous pure water evaporation completion. In contrast, the heat flow temperature curves of pure water in STHE are broader with lower peaks, further illustrating the distinction between the two types of water evaporation. This observation suggests that the evaporation behavior of water in STHE differs from that of bulk water. The quantitative evaporation enthalpies of pure water and the saturated STHE in pure water were calculated to be 2224 J g^−1^, which is close to its theoretical value,^[^ ^52^ ^]^ and 1772 J g^−1^ respectively (Figure 5b,c). The reduced evaporation enthalpy of water in STHE could be attributed to the electrostatic interactions between the charged groups on the SA‐TA chains and adjacent water molecules, as well as the hydrogen bonding between the ‐OH groups on the SA and TA chains and water molecules within the interpenetrating network composed of SA‐TA. The hydroxyl groups in SA‐TA networks could form strong hydrogen bonds with water molecules, disturbing and weakening hydrogen bonds within water clusters so that the phase change of the water phase took place more easily.^[^ ^53^ ^]^ The dark experiments were carried out to calculate the equivalent evaporation enthalpy of STHE in pure water (details shown in Figure S20 and Note S1, Supporting Information). The evaporation enthalpy of the saturated STHE in water from the dark experiment was 1330 J g^−1^, lower than that calculated by DSC due to its full dehydration.^[^ ^54^, ^55^ ^]^

As depicted in Figure 5c, the water within the porous network of STHE can be categorized into three types: 1) bound water that is tightly associated with the surface of SA‐TA polymer chains; 2) intermediate water (IW), which exhibits weakened interactions with functional groups of SA‐TA molecular chains and surrounding water molecules; 3) free water (FW), which solely interacts with other surrounding water molecules. Raman spectroscopy is employed for further analysis of the three distinct types of water molecules present in the STHE. As shown in Figure 5d,e, the Raman spectra of the STHEs are fitted with four peaks using Gaussian functions, located at ≈3100–3700 cm^−1^, respectively in the region of O─H stretching.^[^ ^10^, ^56^ ^]^ Each water molecule can engage in the formation of four hydrogen bonds with other water molecules, utilizing two protons and two lone electron pairs. The peaks observed at 3212 and 3316 cm^−1^ correspond to hydrogen bonding and are associated with free water. Intermediate water, characterized by weakly or non‐hydrogen bonded water molecules where the hydrogen bonds are partially or entirely disrupted, is assigned to the peaks at 3514 and 3630 cm^−1^. The ratio of IW to FW was 1.42, which was much higher than that of pure water (0.44) in the STHE network (Figure 5f). The presence of IW facilitates water molecules to evaporate at a lower energy state, resulting in water activation and reducing the evaporation enthalpy. This mechanism enhances the evaporation performance of STHE.

### 2.6 Applications and Outdoor Testing of STHE

**Figure 6:**
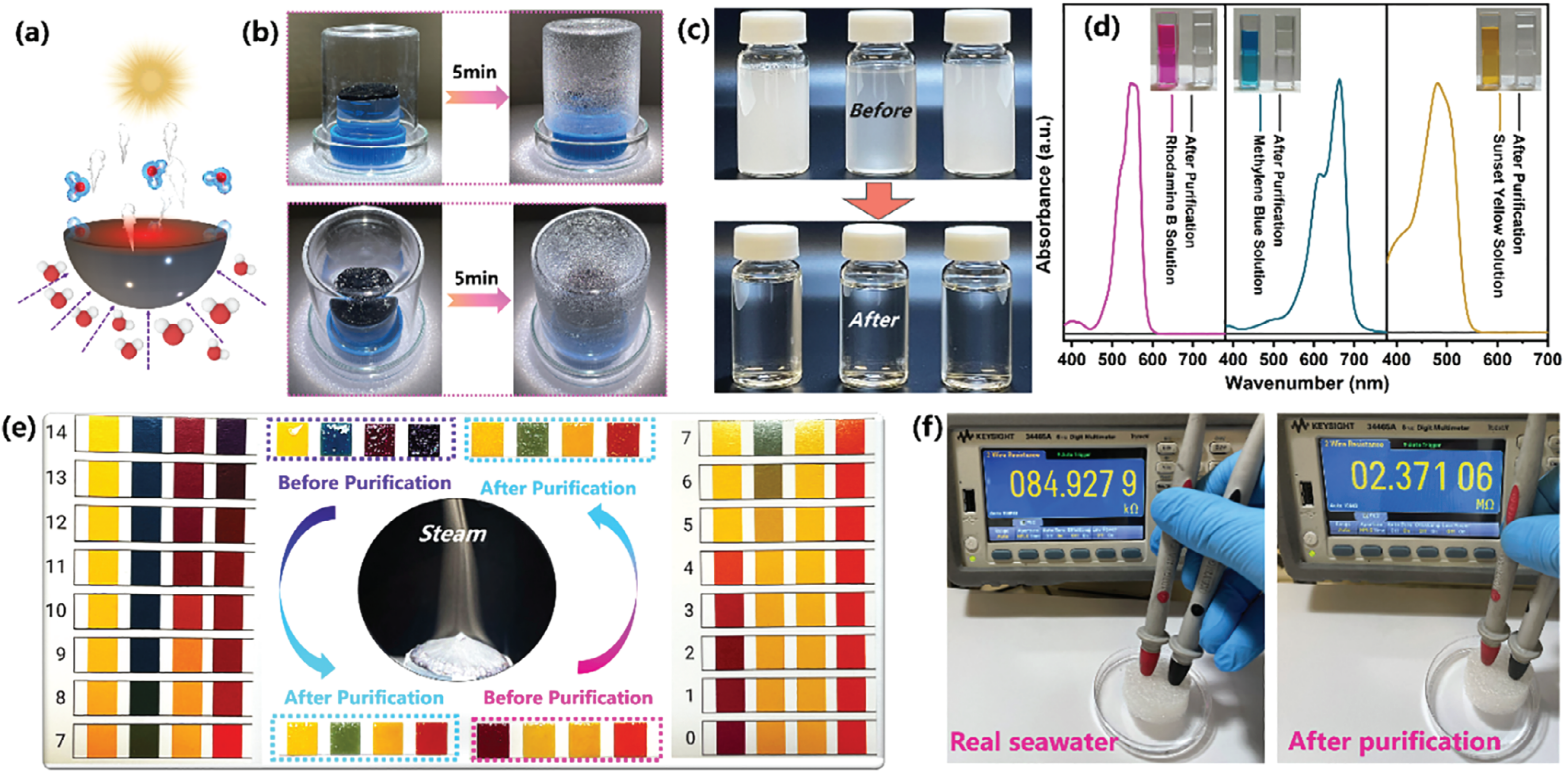
Applications of Interfacial Solar‐driven STHE. a) Schematic diagram illustrating solar‐driven water evaporation using the STHE; b) Photographs of the STHE under one sun illumination; c) Photographs of oil‐water emulsion (vegetable oil, silicone oil, and tween 80 in water) before and after treatment by STHE; d) Ultraviolet absorbance curves of Rhodamine B, Methylene Blue and Sunset Yellow solution before and after purification by STHE; e) Photographs depicting pH changes in acidic and alkaline aqueous solutions before and after purification using the STHE; f) Electrical resistance measurements of real seawater before and after desalination by STHE.

The illustration of the STHE under light was for evaporation depicted in **Figure** **6**a, where the STHE acts as a “pump” for providing continuous unidirectional water supply for evaporation. Under one sun irradiation, numerous tiny water droplets condensed on the glass cover within 5 min, as clearly depicted in Figure 6b. Colorless and clean water from milky oil‐in‐water emulsions were collected through this system and processed by the STHE, demonstrating the complete purification of the emulsions by the STHE (Figure 6c). This characteristic, combined with the previously demonstrated underwater oil repellence (Figure 3d–f), affirms the outstanding and reliable oily water purification of STHE. As shown in Figure S21 (Supporting Information), the evaporation rate of the STHE exhibited minimal decline and remained above 2.0 kg m^−^^2^ h in the oil‐seawater emulsion, highlighting its exceptional multifunctionality and stable performance in mixed contaminant conditions. Furthermore, the purified water by STHE from Rhodamine B, Methylene Blue, and Sunset Yellow aqueous solution became colorless and the strong characteristic UV absorption peaks almost vanished (Figure 6d), demonstrating the ≈100% rejection rate of organic dyes. Additionally, the pH values of condensed water after the purification of HCl and NaOH aqueous solutions approached 7 (Figure 6e), underscoring its significant promise in treating wastewater under rigorous conditions. After 1 day of exposure to acidic and alkaline wastewater, the FT‐IR spectra of the treated STHE showed no significant changes compared to the original sample (shown in Figure S22, Supporting Information), highlighting its exceptional chemical stability under harsh conditions.

To further confirm the versatility of STHE for real seawater desalination, seawater was collected from the nearest sea, Port Phillip Bay, Victoria, Australia, for solar‐driven desalination experiments (solar data shown in Figure S23, Supporting Information). The aqueous electrical resistance of real seawater was 84.93 KΩ, whereas that of the desalinated water significantly decreased to 2.37 MΩ (Figure 6f), highlighting the impressive desalination performance of STHE. The quality of the collected water by photothermal desalination is crucial for practical applications. Thus, the inductively coupled plasma mass spectrometry (ICP‐MS) was used to quantitate the collected water quality by photothermal desalination of STHE. As shown in Figure S24 (Supporting Information), the concentrations of four types of ions (Ca^2^⁺, Mg^2^⁺, Na⁺, and K⁺) in the collected water from the STHE meet the World Health Organization (WHO) standards, confirming its remarkable reliability of photothermal desalination. Even after one month of immersion in seawater, the structure of STHE remained intact, and the real seawater evaporation rate of STHE was consistently maintained at 2.1 kg m^−2^ h, with no significant decrease compared to its initial rate (shown in Figure S25, Supporting Information). This result highlights the remarkable structural stability and reliable evaporation performance of the STHE over an extended period. These observations provide valuable insights into the long‐term stability of the as‐prepared STHE, confirming its resistance to degradation and consistent performance under extended seawater exposure. In summary, this biomimetic STHE has showcased its potential as a highly efficient solar‐driven treatment of a range of water‐oily, dyed, and brine water, offering promising advancements in these critical domains.

**Figure 7:**
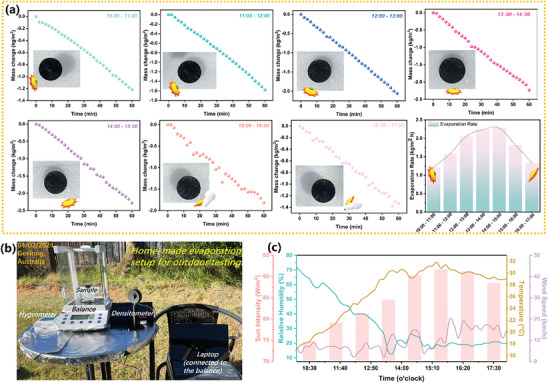
Outdoor Water Evaporation performance of STHE. a) Evaporation rates of the STHE corresponding to varying solar densities observed outdoors from 10:00–17:00; b) Optical image of the home‐made setup used for outdoor testing; c) Environmental parameters (relative humidity, temperature, and wind speed) measured during the daytime of outdoor testing.

A prototype outdoor solar‐driven water evaporation system, employing STHE, was engineered to further validate the practical use of the STHE, operating between 10:00 and 17:00, on March 4, 2024, in Geelong, Victoria, Australia (**Figure** **7**b). During the experiment conducted from 10:00 to 17:00 under natural sunlight, solar steam generation rates and environmental parameters (solar density, relative humidity, and wind speed) were continuously monitored and recorded in real‐time (Figure 7a,c). The solar intensity peaked at ≈93 mW cm^2^ ≈15:00. In contrast to the constant conditions (100 mW cm^−2^) typically found in laboratory settings, the actual outdoor environment experiences numerous fluctuations that can significantly impact the evaporation rates. Therefore, the fluctuation trends in solar steam generation rates of STHE (Figure 7a) closely align with the variations in sunlight intensity (Figure 7c). Environmental humidity can affect the performance of the evaporator, as higher humidity levels might reduce the evaporation rate by slowing down water vapor diffusion into the surrounding air (Figure 7c). Nonetheless, it was estimated that the STHE could generate ≈12.8 kg m^−2^ of freshwater daily, highlighting its reliable and excellent outdoor evaporation performance and promising potential for mitigating worldwide freshwater scarcity. Given the scope and objectives of this work, specific biocompatibility and biosafety experiments^[^ ^57^, ^58^ ^]^ on the STHE were not conducted. Nevertheless, such assessments would offer an additional, meaningful perspective and are well‐suited for a separate, in‐depth study in the future. Due to the low density of STHE, the evaporator easily floats on the water surface (shown in Figure 3d). Its hemispherical structure, similar to a tumbler toy, provides excellent stability on the water surface even under shaking states, simulating natural wind and wave disturbances,^[^ ^59^, ^60^ ^]^ as depicted in Figure S26 (Supporting Information). This stability highlights its great potential for practical applications.

## 3 Conclusion

In summary, taking inspiration from nature, we have proposed a multidirectional freezing strategy (MDF) for crafting a hemispheric STHE incorporated tapered channels aligned in a spatially centripetal manner. These alignments exhibited anti‐gravity and unidirectional water transfer properties driven by Laplace asymmetric pressure effects. To enable reliable solar‐driven water evaporation, the device is engineered with a robust and stable photothermal layer by chelation of tannic acid (TA) with Fe^3+^ (MPN). It is the first time to combine well‐designed tapered channels for unidirectional water transfer and a robust MPN layer for high‐efficiency light‐to‐heat conversion into an all‐in‐one system. Consequently, the STHE with biomimetic channels can provide a new perspective to effectively manage water supply in one direction within a solar evaporator. This contributes to preventing water backflow so that downside conduction heat loss is minimized to enhance energy efficiency. Thanks to the design merits of this STHE with reduced water evaporation enthalpy, the evaporator achieved a high evaporation rate of ≈2.26 kg m^−2^ h^−1^ under 1 sun illumination. Moreover, the densely ionic channels oriented in the centripetal direction result in a significant osmotic pressure, facilitating the exclusive pumping of water upward while repelling the ions in saline water. The STHE retained its structure and steady evaporation rate of 2.10 kg m^−2^ h^−1^ after one month of seawater immersion, confirming its stability and resistance to degradation. Even with a salt concentration as high as 15.0 wt.%, the surface of the STHE did not accumulate salt crystals, and there was only a slight decrease in evaporation rates. The hemispheric structure and superhydrophilicity of the STHE were demonstrated to be conducive to anti‐oil‐repellence. As a proof of concept, this STHE evaporator effectively purifies various types of water, including seawater, oily wastewater, dye wastewater, acid, and alkali wastewater. Thus, this work demonstrates a promising strategy to enhance water evaporation rates and efficiently generate freshwater from various water sources, presenting a sustainable and reliable solution to tackle global water scarcity.

## 4 Experimental Section

Experimental details are given in the Supporting Information.

## Conflict of Interest

The authors declare no conflict of interest.

## Author Contributions

Y.B. performed methodology, conceptualization, investigation, characterization, wrote and prepare the final manuscript. X.L. performed conceptualization, software analysis, wrote reviewed, and edited the final manuscript. W.L. wrote reviewed, and edited the final manuscript. H.Y. performed conceptualization, supervision, wrote reviewed, and edited the final manuscript, funding acquisition. W.X. performed conceptualization, supervision. Q.H. performed characterization. J. L. performed conceptualization, supervision, wrote reviewed, and edited the final manuscript, funding acquisition.

## Supporting information



Supporting Information

## Data Availability

The data that support the findings of this study are available in the supplementary material of this article.

## References

[1] N. Hanikel , M. S. Prévot , O. M. Yaghi , Nat. Nanotechnol 2020, 15, 348. 32367078 10.1038/s41565-020-0673-x

[2] C. J. Vorosmarty , P. Green , J. Salisbury , R. B. Lammers , Science 2000, 289, 284. 10894773 10.1126/science.289.5477.284

[3] A. V. Dudchenko , C. Chen , A. Cardenas , J. Rolf , D. Jassby , Nat. Nanotechnol. 2017, 12, 557. 28553963 10.1038/nnano.2017.102

[4] X. Hao , H. Yao , P. Zhang , Q. Liao , K. Zhu , J. Chang , H. Cheng , J. Yuan , L. Qu , Nat. Water 2023, 1, 982.

[5] T. Liu , M. S. Mauter , Joule 2022, 6, 1199.

[6] H. B. Park , J. Kamcev , L. M. Robeson , M. Elimelech , B. D. Freeman , Science 2017, 356, eaab0530. 28619885 10.1126/science.aab0530

[7] M. Gao , L. Zhu , C. K. Peh , G. W. Ho , Energy Environ. Sci. 2019, 12, 841.

[8] P. Tao , G. Ni , C. Song , W. Shang , J. Wu , J. Zhu , G. Chen , T. Deng , Nat. Energy 2018, 3, 1031.

[9] W. Ma , T. Lu , W. Cao , R. Xiong , C. Huang , Adv. Funct.Mater. 2023, 33, 2214157.

[10] F. Zhao , X. Zhou , Y. Shi , X. Qian , M. Alexander , X. Zhao , S. Mendez , R. Yang , L. Qu , G. Yu , Nat. Nanotechnol. 2018, 13, 489. 29610528 10.1038/s41565-018-0097-z

[11] L. Zhou , Y. Tan , J. Wang , W. Xu , Y. Yuan , W. Cai , S. Zhu , J. Zhu , Nat. Photonics 2016, 10, 393.

[12] F. Seidi , W. Jiang , Z. Yu , C. Deng , J. Bioresour. Bioprod. 2024, 9, 243.

[13] Y. Bu , Y. Zhou , W. Lei , L. Ren , J. Xiao , H. Yang , W. Xu , J. Li , J. Mater. Chem. A 2022, 10, 2856.

[14] C. Cai , Y. Sun , Y. Chen , Z. Wei , Y. Wang , F. Chen , W. Cai , J. Ji , Y. Ji , Y. Fu , J. Bioresour. Bioprod. 2023, 8, 421.

[15] X. Liu , Y. Tian , F. Chen , A. Caratenuto , J. A. DeGiorgis , M. ELSonbaty , Y. Wan , R. Ahlgren , Y. Zheng , Adv. Funct. Mater. 2021, 31, 2100911.

[16] Z. Liu , B. Wu , B. Zhu , Z. Chen , M. Zhu , X. Liu , Adv. Funct. Mater. 2019, 29, 1905485.

[17] M. Zou , Y. Zhang , Z. Cai , C. Li , Z. Sun , C. Yu , Z. Dong , L. Wu , Y. Song , Adv. Mater. 2021, 33, 2102443. 10.1002/adma.202102443 34288134

[18] Z. Wang , X. Wu , F. He , S. Peng , Y. Li , Adv. Funct. Mater. 2021, 31, 2011114.

[19] F. Li , N. Li , S. Wang , L. Qiao , L. Yu , P. Murto , X. Xu , Adv. Funct. Mater. 2021, 31, 2104464.

[20] X. Li , J. Li , J. Lu , N. Xu , C. Chen , X. Min , B. Zhu , H. Li , L. Zhou , S. Zhu , Joule 2018, 2, 1331.

[21] L. Wang , H. Su , Z. Zhang , J. Xin , H. Liu , X. Wang , C. Yang , X. Liang , S. Wang , H. Liu , Angew. Chem., Int. Ed. 2023, 135, e202314185. 10.1002/anie.202314185 37858292

[22] W. Cao , W. Ma , T. Lu , Z. Jiang , R. Xiong , C. Huang , J. Colloid Interface Sci. 2022, 608, 164. 34626964 10.1016/j.jcis.2021.09.194

[23] Q. Fan , T. Lu , Y. Deng , Y. Zhang , W. Ma , R. Xiong , C. Huang , Sep. Purif. Technol. 2022, 297, 121445

[24] H. Ren , M. Tang , B. Guan , K. Wang , J. Yang , F. Wang , M. Wang , J. Shan , Z. Chen , D. Wei , Adv. Mater. 2017, 29, 1702590. 10.1002/adma.201702590 28833544

[25] L. Wu , Z. Dong , Z. Cai , T. Ganapathy , N. X. Fang , C. Li , C. Yu , Y. Zhang , Y. Song , Nat. Commun. 2020, 11, 521 31988314 10.1038/s41467-020-14366-1 PMC6985111

[26] P. Xiao , J. Gu , C. Zhang , F. Ni , Y. Liang , J. He , L. Zhang , J. Ouyang , S.‐W. Kuo , T. Chen , Nano Energy 2019, 65, 104002.

[27] L. Li , N. He , B. Jiang , K. Yu , Q. Zhang , H. Zhang , D. Tang , Y. Song , Adv. Funct. Mater. 2021, 31, 2104380.

[28] H. Liu , R. Jin , S. Duan , Y. Ju , Z. Wang , K. Yang , B. Wang , B. Wang , Y. Yao , F. Chen , Small 2021, 17, 2100969. 10.1002/smll.202100969 33938137

[29] T. A. Cooper , S. H. Zandavi , G. W. Ni , Y. Tsurimaki , Y. Huang , S. V. Boriskina , G. Chen , Nat. Commun. 2018, 9, 5086. 30538234 10.1038/s41467-018-07494-2 PMC6290071

[30] V. Kashyap , A. Al‐Bayati , S. M. Sajadi , P. Irajizad , S. H. Wang , H. Ghasemi , J. Mater. Chem. A 2017, 5, 15227.

[31] X. Dong , Y. Si , C. Chen , B. Ding , H. Deng , ACS Nano 2021, 15, 12256. 34151558 10.1021/acsnano.1c04035

[32] G. Ni , S. H. Zandavi , S. M. Javid , S. V. Boriskina , T. A. Cooper , G. Chen , Energy Environ.Sci. 2018, 11, 1510.

[33] C. Shen , Y. Zhu , X. Xiao , X. Xu , X. Chen , G. Xu , ACS Appl. Mater. Interfaces 2020, 12, 35142. 32634301 10.1021/acsami.0c11332

[34] Y. Liang , D. Wang , H. Yu , X. Wu , Y. Lu , X. Yang , G. Owens , H. Xu , Sci. Bull. 2024, 69, 3590. 10.1016/j.scib.2024.09.015 39353816

[35] X. Liu , F. Chen , Y. Li , H. Jiang , D. D. Mishra , F. Yu , Z. Chen , C. Hu , Y. Chen , L. Qu , Adv. Mater. 2022, 34, 2203137. 10.1002/adma.202203137 35839320

[36] Y. Gu , X. Mu , P. Wang , X. Wang , Y. Tian , A. Wei , J. Zhang , Y. Chen , Z. Sun , J. Zhou , Glob. Chall. 2021, 5, 2000063. 33437526 10.1002/gch2.202000063 PMC7788587

[37] Y. Wang , X. Sun , S. Tao , Environ. Sci. Technol. 2020, 54, 16240. 33263990 10.1021/acs.est.0c05449

[38] Y. Tang , Q. Miao , S. Qiu , K. Zhao , L. Hu , J. Eur. Ceram. Soc. 2014, 34, 4077.

[39] L. Pu , H. Ma , J. Dong , C. Zhang , F. Lai , G. He , P. Ma , W. Dong , Y. Huang , T. Liu , Nano Lett. 2022, 22, 4560. 35583326 10.1021/acs.nanolett.2c01486

[40] C. Wang , X. Chen , B. Wang , M. Huang , B. Wang , Y. Jiang , R. S. Ruoff , ACS Nano 2018, 12, 5816. 29757617 10.1021/acsnano.8b01747

[41] Y. Bu , X. Li , W. Lei , H. Su , H. Yang , W. Xu , J. Li , J. Mater. Chem. A 2023, 11, 15147.

[42] P. Zhu , L. Wang , Chem. Rev. 2021, 122, 7010. 34918913 10.1021/acs.chemrev.1c00530

[43] W. Zhang , Z.‐Y. Yang , R.‐C. Tang , J.‐P. Guan , Y.‐F. Qiao , J. Cleaner Prod. 2020, 250, 119545.

[44] C. Zhang , Z. Chen , Z. Xia , R. Z. Waldman , S.‐L. Wu , H.‐C. Yang , S. B. Darling , Environ. Sci.: Water Res. Technol. 2020, 6, 911.

[45] W. Ma , W. Cao , M. Cui , Q. Fan , R. Xiong , C. Huang , J. Mater. Chem. A 2024, 12, 13520.

[46] Z. Zhao , C. Wang , D. Wei , Q. Hu , P. Tan , F. Wang , Y. Xie , W. Zhang , J. Zhang , Small 2024, 20, 2402482. 10.1002/smll.202402482 38855997

[47] H. Ejima , J. J. Richardson , K. Liang , J. P. Best , M. P. van Koeverden , G. K. Such , J. Cui , F. Caruso , Science 2013, 341, 154. 23846899 10.1126/science.1237265

[48] J. Guo , B. L. Tardy , A. J. Christofferson , Y. Dai , J. J. Richardson , W. Zhu , M. Hu , Y. Ju , J. Cui , R. R. Dagastine , Nat. Nanotechnol 2016, 11, 1105. 27723730 10.1038/nnano.2016.172

[49] H. Song , Y. Liu , Z. Liu , M. H. Singer , C. Li , A. R. Cheney , D. Ji , L. Zhou , N. Zhang , X. Zeng , Adv. Sci. 2018, 5, 1800222. 10.1002/advs.201800222 PMC6096986 30128237

[50] Z. Y. Wang , Y. J. Zhu , Y. Q. Chen , H. P. Yu , Z. C. Xiong , Small 2023, 19, 2206917. 10.1002/smll.202206917 36793253

[51] J. Zeng , Q. Wang , Y. Shi , P. Liu , R. Chen , Adv.Energy Mater 2019, 9, 1900552.

[52] J. Zhao , A. Chu , J. Chen , P. Qiao , J. Fang , Z. Yang , Z. Duan , H. Li , Chem. Eng. J. 2024, 485, 150118.

[53] Z. Yu , P. Wu , Adv. Mater. Technol. 2020, 5, 2000065.

[54] Y. Wei , W. Li , S. Zhang , J. Yu , Y. Tang , J. Wu , S. Yu , Adv. Funct. Mater. 2024, 34, 2401149.

[55] X. Zhou , F. Zhao , Y. Guo , B. Rosenberger , G. Yu , Sci. Adv. 2019, 5, eaaw5484. 31259243 10.1126/sciadv.aaw5484 PMC6599166

[56] C. Yang , B. H. Ko , S. Hwang , Z. Liu , Y. Yao , W. Luc , M. Cui , A. S. Malkani , T. Li , X. Wang , Sci. Adv. 2020, 6, eaaz6844. 32494647 10.1126/sciadv.aaz6844 PMC7182425

[57] Q. Qu , J. Zhang , X. Chen , H. Ravanbakhsh , G. Tang , R. Xiong , B. B. Manshian , S. J. Soenen , F. Sauvage , K. Braeckmans , ACS. Sustain. Chem. Eng. 2020, 9, 387.

[58] T. Lu , H. Liang , W. Cao , Y. Deng , Q. Qu , W. Ma , R. Xiong , C. Huang , J. Colloid. Interface Sci. 2022, 608, 2860. 34802769 10.1016/j.jcis.2021.11.017

[59] W. Ma , W. Cao , M. Cui , H. Lu , R. Xiong , C. Huang , Chem. Eng. J. 2023, 478, 147404.

[60] W. Ma , T. Lu , W. Cao , R. Xiong , C. Huang , Adv. Funct. Mater. 2023, 33, 2214157.

