# Supplementary material for: Advanced Bionic 3D Interfacial Solar Steam Generator With One‐way Water Supply for Highly Efficient Desalination and Oil‐Fouling Prevention
Source: Adv Sci (Weinh). 2024 Dec 25;12(7):2412545. doi: 10.1002/advs.202412545 (PMC11831532; doi:10.1002/advs.202412545)
Supplement: Supplementary file 1 — Supporting Information [file ADVS-12-2412545-s001.docx]

**Advanced Bionic 3D Interfacial Solar Steam Generator with One-way Water Supply for Highly Efficient Desalination and Oil-Fouling Prevention**

Yiming Bu^1^, Xin Li^1^, Weiwei Lei^1^, Hongjun Yang^2*^, Weilin Xu^2^, Qi Han^3^, Jingliang Li^1*^

1. Institute for Frontier Materials, Deakin University, Geelong, VIC 3216, Australia.

2. Key Laboratory of Green Processing and Functional New Textile Materials of Ministry of Education, Wuhan Textile University, Wuhan 430200, China.

3. School of Science, RMIT University, City Campus, Melbourne, VIC 3000, Australia.

E-mail: h_j.yang@yahoo.com (H.Y.); jingliang.li@deakin.edu.au (J.L.)

**This PDF file includes:**

Experimental Section

Note S1

Note S2

Note S3

Note S4

Figure S1-S26

Reference

**Experimental Section**

**Materials, and chemicals**

Sodium alginate, tannic acid, ferric chloride, sodium hydroxide, hydrogen chloride, Rhodamine B, Methylene Blue, Sunset Yellow, tween 80 and silicone oil were supplied by Sigma-Aldrich and all regents were used without any further purification. Vegetable oil was bought from local supermarket. The copper moulds with hemispheric depression were manufactured by a local factory.

**Material characterization**

The surface morphology of carbon fibre was characterized by scanning electron microscopy (SEM) (Sigma 500, Zeiss, Germany). UV-Vis-NIR spectra were recorded by a UV-2600 Spectrophotometer (Shimadzu). Infrared Radiation (IR) camera (FLIR-E6390, FLIR, Sweden) was used to characterize temperature distributions. Fourier-transform infrared (FT-IR) spectra of the sorbents were obtained using an FT-IR spectrometer (Is 50, ThermoFisher Scientific). The 3D optical microscopy images of the samples were obtained via a 3D laser microimaging system (VK-150 K, Keyence). Specific heat flow was tested by DSC (Q 200, TA Instruments). Raman spectroscopy was characterized by a Via-Reflex spectrometer (Renishaw) with a laser of 532 nm wavelength. 3D structure of STHE scaffold was imaged by a micro-CT equipped with tungsten X-ray source from X-rayWorx operating with a voltage of 50 kV and power of 2 W.

**Fabrication of 3D STH evaporator**

Defoamed aqueous dispersion of SA (2.0 wt %)-TA (1.0 wt %) mixture was transferred into the mould for multidirectional freeze-casting (MDF). After sublimation of the ice pillars, a freeze-dried SA-TA Hemisphere (STH) aligned with spatially centripetal conical channels was obtained. For comparison, different STHs with variable concentrations of SA (1.0, 1.5, 2.0 and 2.5 wt %) and constant concentration of TA were prepared by the same method. The TA-Fe^3+^ metal polyphenol network (MPN) layer was engineered by uniformly spraying 1 mL 15% ferric chloride aqueous solution onto the surface of as-prepared STH, where the stable black TA-Fe^3+^ metal polyphenol network layer was rapidly formed. That is denoted as SA-TA Hemispheric Evaporator (STHE).

**Solar-driven steam generation tests**

Simulated sunlight was provided by a xenon lamp (CELHXF300, Education Au-light Co Beijing) with an optical filter for the standard AM 1.5G spectrum. The intensity of solar irradiation was controlled from 0.5-2.0 W cm^-2^. An electronic balance with an accuracy of 0.0001 g (Sartorius, SQP, QUNITIX224-1CN) was used to record the weight change at regular intervals during the test. An STHE with lotus seedpods-like water supply (bottom-to-up) was exposed to simulated sunlight for solar-driven desperation in a room temperature around 25 °C and 45 % RH.

**Note S1. Estimation of equivalent evaporation enthalpy.**

Experiments in a dark environment were conducted to calculate the equivalent evaporation enthalpy of STHE, as the actual evaporation process with an evaporator is different from the fully dehydrated condition measured by DSC. The same superficial area of water and STHE were synchronously set in a closed container with a supersaturated MgCl_2_ solution at 25 ºC and under ambient air pressure. By evaporating the water with the identical power input (*U_in_*), the equivalent evaporation enthalpy (*∆H_equ_*) of the water in the evaporator can be estimated as follow:

*U_in_ = ∆H_water_·m_water_ = ∆H_equ_·m_sample_* (S1)

where *∆H_water_* and *m_water_* are the evaporation enthalpy and mass change of bulk water; *m_sample_* is the mass change of STHE-water. The energy efficient is also calculated based on *∆H_equ_* of corresponding evaporator.

The evaporation enthalpy of pure water at different temperature was calculated by the following Equation:

Δ𝐻_0_ = 𝐶_1_ + 𝐶_2_𝑇 + 𝐶_3_𝑇^1.5^ + 𝐶_4_𝑇^2.5^ + 𝐶_5_𝑇^3^  (S2)

where 𝐶_1_ = 2500.304, 𝐶_2_ = -2.2521025, 𝐶_3_ = -0.021465847, 𝐶_4_ = 3.1750136×10–4, 𝐶_5_ = -2.8607959×10­^–5^ are constants, and 𝑇 is temperature (°C). Thus, the Δ𝐻_water_ of pure water at 25 °C was 2442 kJ kg^–1^. Thus, the calculated evaporation enthalpy of STHE in water from dark experiment is 1330 kJ kg^–1^.

**Note S2. Detailed calculation of the solar-to-vapor efficiency of STHE**

The evaporation efficiency could be calculated by the following equation:

$\eta=(\dot{m}\cdot h_{lv})/(C_{opt}\cdot q_{i})$ (S3)

where $\dot{m}$ (kg/m^2^ h) is the water evaporation of STHE, $h_{lv}$ (J/g) represents the equivalent vaporization enthalpy, $C_{opt}$ is the optical concentration of sunlight irradiance, and $q_{i}$ is the power of 1 sun irradiance (1 kW m^–2^). To calculate the equivalent evaporation enthalpies of the hydrogels, comparative experiments were conducted in a dark environment to ensure consistent energy input (𝑈_𝑖𝑛_), which is explained in Note S1. Thus, the calculated solar evaporation efficiency of the STHE hydrogel, after subtracting the corresponding dark evaporation, is 83.5% based on the equivalent enthalpies.

**Note S3. Detailed calculation of the conductive heat loss of STHE**

*Q_cond_* = C m ΔT (S4)

*η_cond =_ Q_cond_ /A q_solar_* (S5)

where *Q_cond_* (J) referred to the heat energy transfer from the evaporator to bulk water, C was the specific heat capacity of pure water (4.2 J g^-1^ K^-1^), m (15g) was the weight of water and ΔT is the temperature change of bulk water under one sun irradiation in 1h.

**Note S4. Detailed calculation of Ion rejection rate of STHE**

The ion rejection rate of the STHE can be calculated using the formula:^[1]^

Ion rejection rate = (1-*W_in_*/*W_s_*)×100% (S6)

where: *W_s_* (g/L) represents the NaCl concentration in the bulk NaCl solution; *W_in_* (g/L)​is the NaCl concentration in the absorbed NaCl solution by the STHE at equilibrium state. The NaCl concentration in the absorbed NaCl solution by the STHE can be determined using the following equations:

*W_in_* = *m_ion_/m_sol_*  (S7)

where: *m_sol_* = *m_s_* - *m_o_* ​, which is the mass of the absorbed NaCl solution; *m_ion_* = *m_d_* - *m_o_​*, which is the mass of NaCl in the absorbed solution. *m_o_* is the mass of the original STHE, *m_s_* is the mass of the STHE saturated in NaCl solution and *m_d_* is the mass of the STHE after being dried. These equations collectively allow for the calculation of the ion rejection rate, demonstrating the efficiency of the STHE in rejecting NaCl.


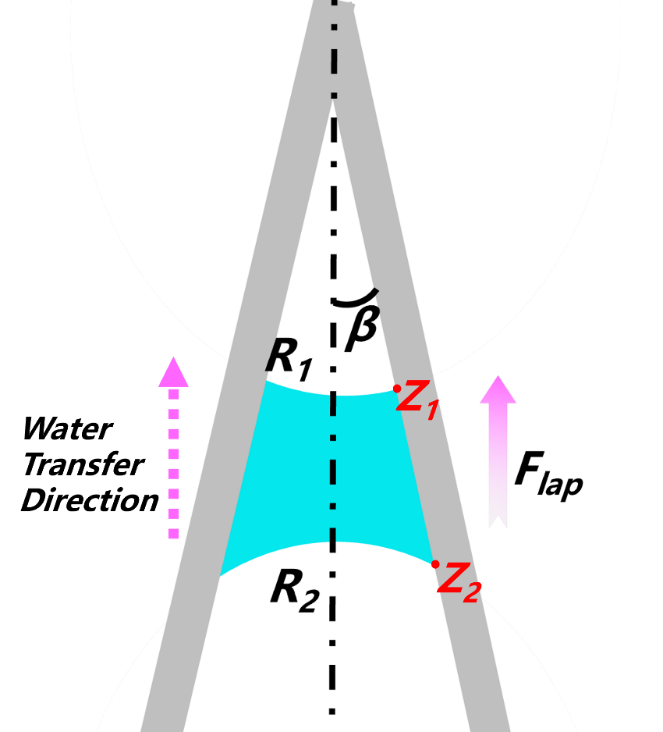


**Figure S1**. Illustration of one-way water transfer inside the tapered channels.

The force of gradient Laplace pressure${(F}_{lap}$) can be estimated as:^[2,3]^

$F_{lap}\boldsymbol{=}\frac{\mathbf{d}\left( \boldsymbol{-}\frac{\boldsymbol{\gamma}_{\mathbf{lv}}}{\boldsymbol{R}} \right)}{\mathbf{d}\boldsymbol{z}}\boldsymbol{=}\frac{\boldsymbol{\gamma}_{\mathbf{lv}}\left( \frac{\mathbf{d}\boldsymbol{R}}{\mathbf{d}\boldsymbol{z}} \right)}{\boldsymbol{R}^{\boldsymbol{2}}}$. (S8)

When $\beta\ll1$, it could be assumed that $R\approx\beta z$ and this driving force of Laplace differential pressure can be mathematically expressed as:^[2,3]^

$F_{lap}\approx\frac{\gamma_{\mathrm{lv}}}{\beta z^{2}}$(S9)

Since $F_{lap}$ is positive, the Laplace pressure is lower on the side of the droplet that is closer to the apex. Consequently, the droplet is propelled towards the intersection by the driving force (shown in **Fig. 2e**). Therefore, by designing such tapered channels, the water droplet inside it could be easily manipulated for one-way transfer.


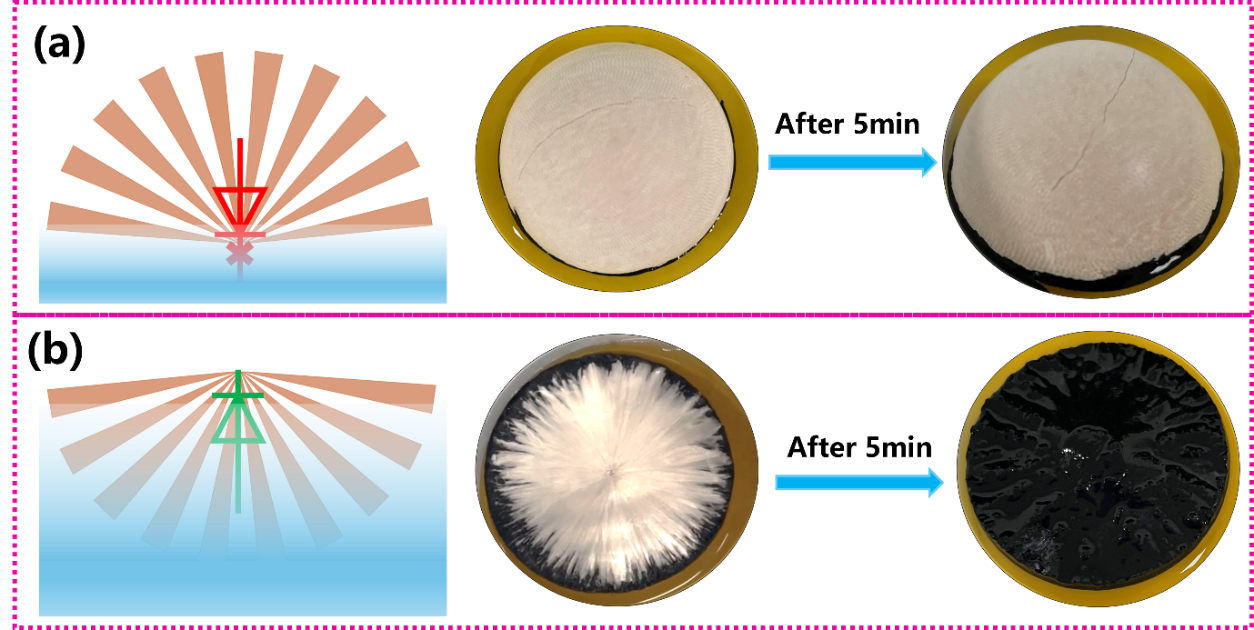


**Figure S2**. Illustration of the anti-gravity one-way water transfer in conical channels of STH: Top to bottom (a) and bottom to top (b) aligned SA-TA hemisphere floating on the ferric aqueous solution.

Two SA-TA hemispheric samples were gently placed onto a ferric aqueous solution in opposite vertical directions (top-to-bottom and bottom-to-top). The colour change resulting from the chelation of Fe³⁺ ions with tannic acid clearly traced the water transfer pathways. As shown in Figure S1(a), no water transfer occurred in the top-to-bottom direction. In contrast, when the hemisphere was oriented bottom-to-top (Figure S1(b)), aligning with the Laplace differential pressure in the conical channels, the aqueous solution was swiftly transferred upwards, highlighting the remarkable one-way water transfer property, even against the force of gravity.


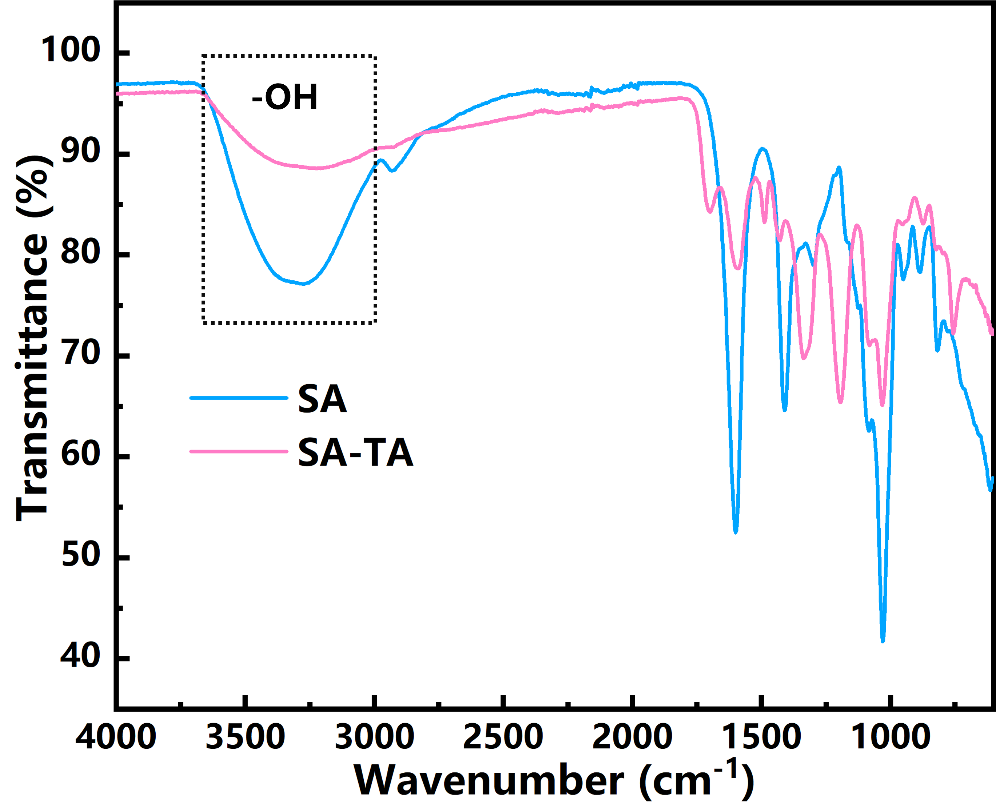


**Figure S3**. Fourier Transform Infrared (FT-IR) spectra of SA and SA-TA.

A distinct and broad band around 3320 cm⁻¹, associated with hydroxyl stretching vibrations, was observed in both SA and SA-TA samples, confirming a high content of -OH groups in both tannic acid and alginate. The reduced peak intensity in the SA-TA samples likely results from interactions between the hydroxyl groups of tannic acid and alginate, suggesting the formation of hydrogen bonds between the two components.


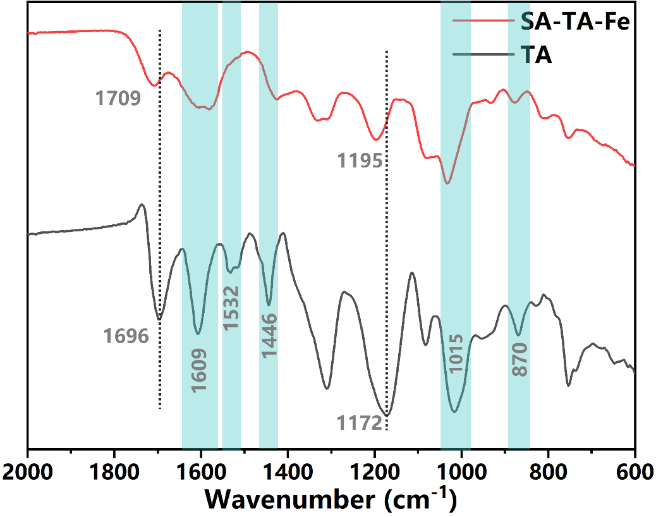


**Figure S4**. Fourier Transform Infrared (FT-IR) spectra of tannic acid (TA) and STHE (SA-TA-Fe).


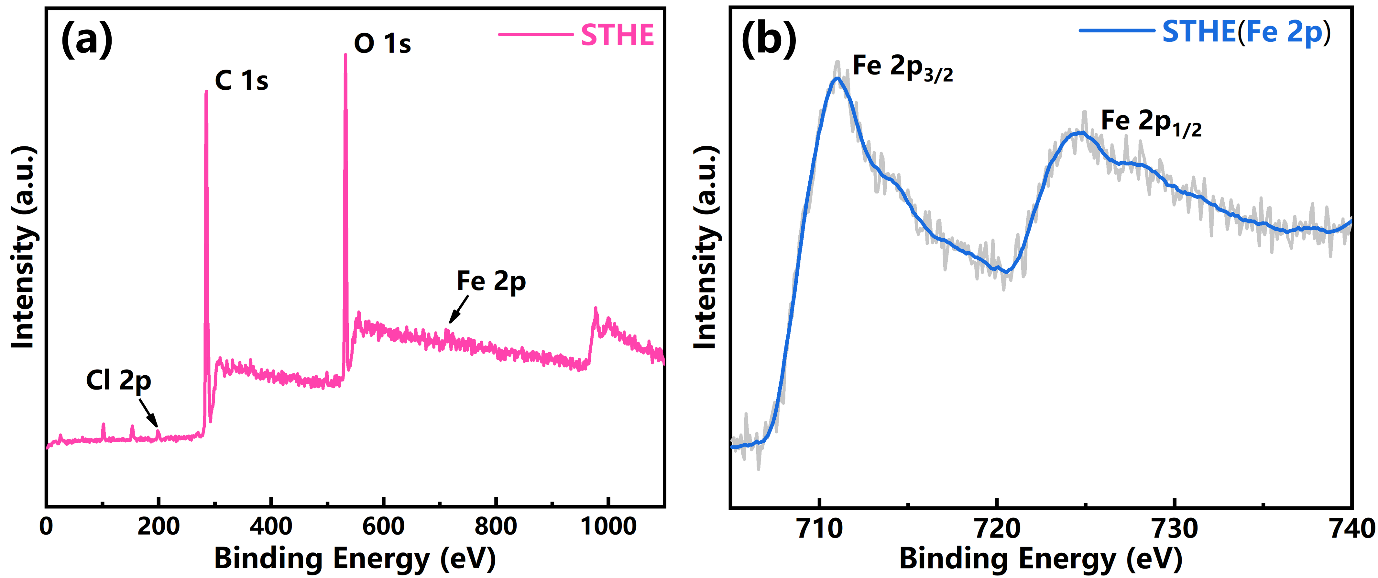


**Figure S5**. (a) X-ray photoelectron spectroscopy (XPS) full peak spectrum of STHE, which exhibits the presence of Fe, Cl, C and O; and (b) high-resolution spectra of Fe 2p of STHE.

**
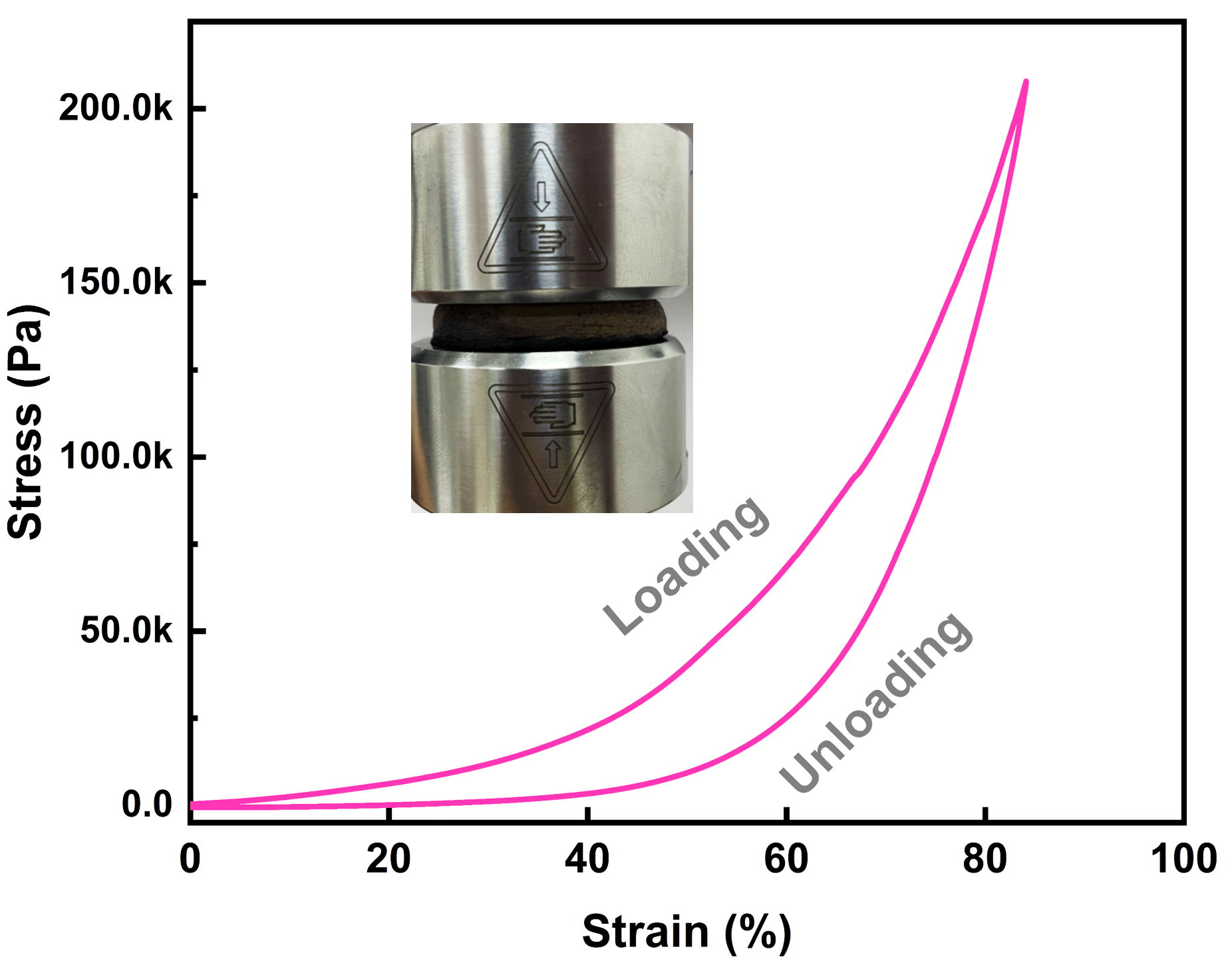
**

**Figure S6**. Stress-strain curve of STHE.


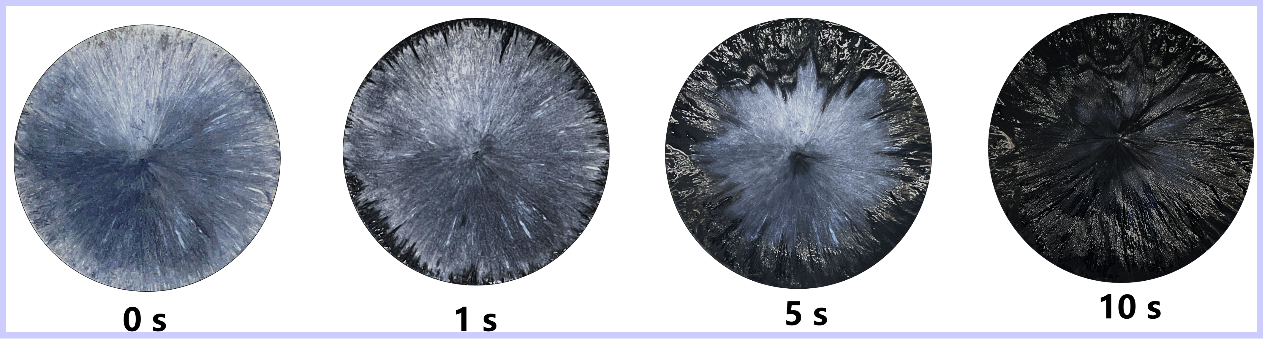


**Figure S7**. Optical images of water absorbing from bottom to up of dry STHE.


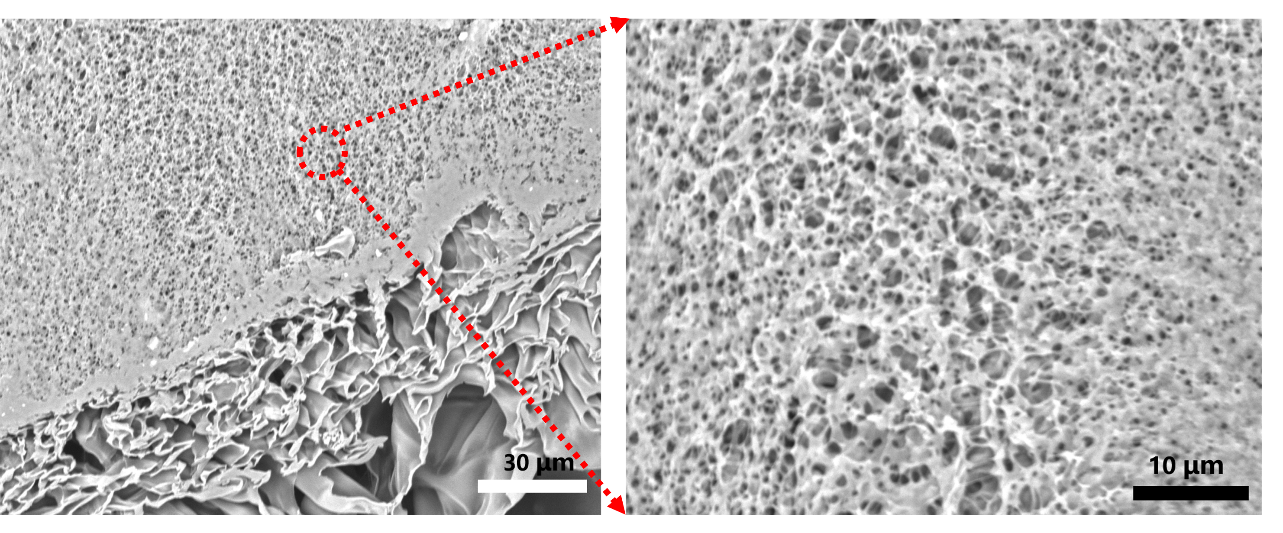


**Figure S8**. SEM images of outer curve of STHE.

**
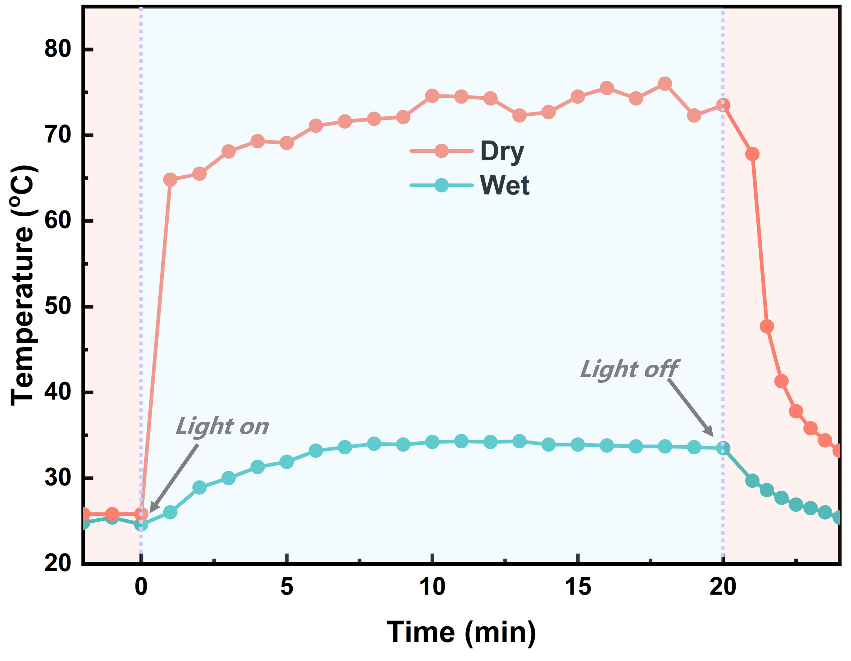
**

**Figure S9**. Surface temperature changes with time under one sun illumination of dry and wet STHE.


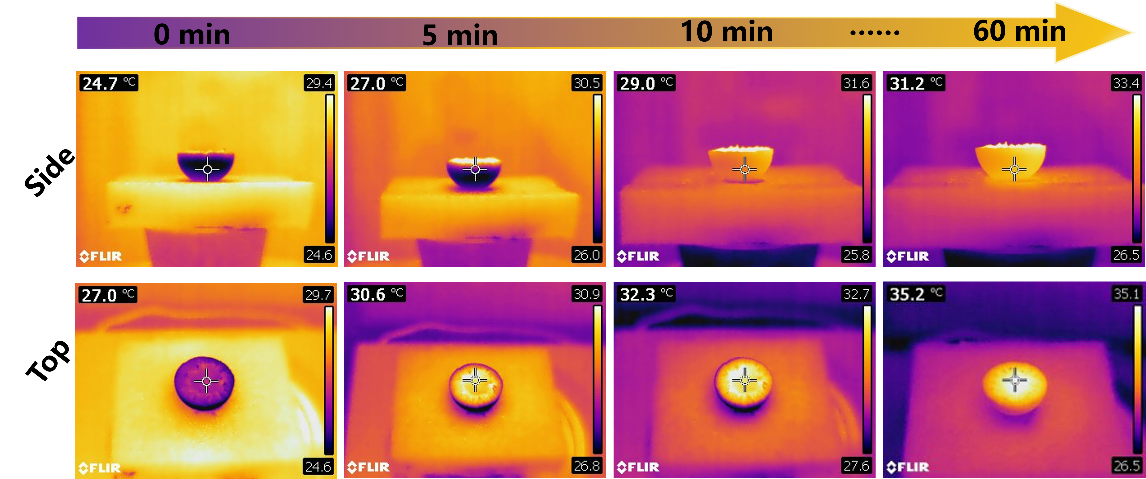


**Figure S10**. Infrared images of STHE under one sun illumination.


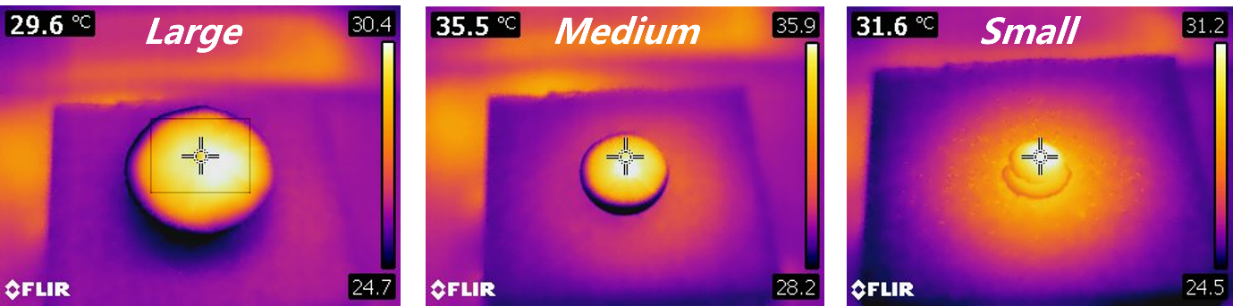


**Figure S11**. Infrared images of STHE with different diameters (Large-4.5 cm, Medium-3.0 cm and Small-1.5 cm).


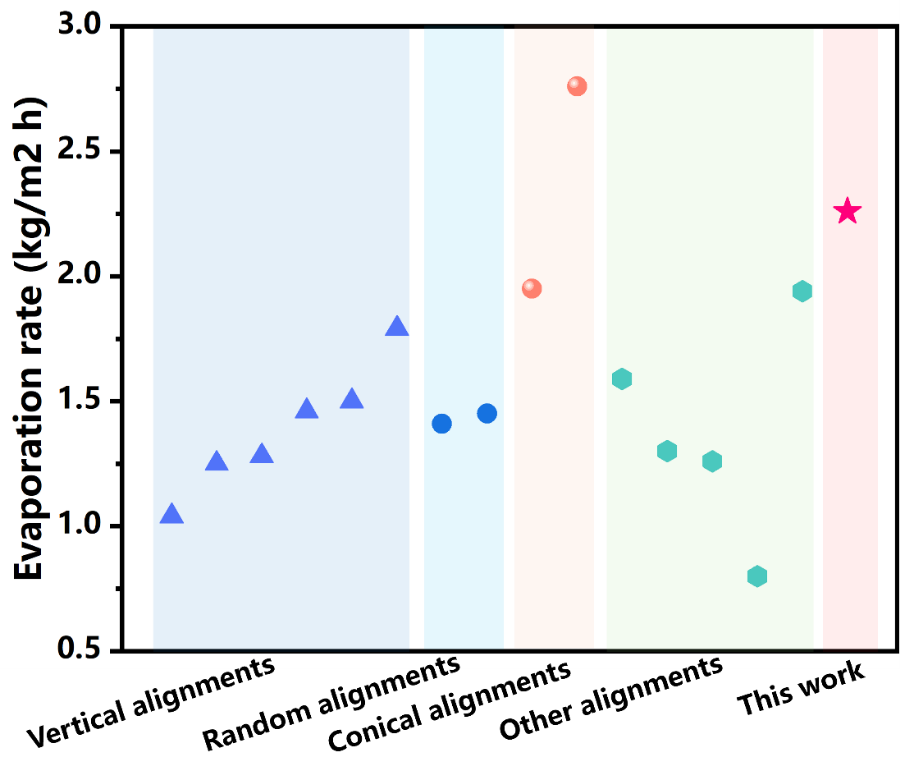


**Figure S12**. Comparison of the evaporation performance of STHE with other state-of-the-art ISSG systems with different alignments.^[4-18]^


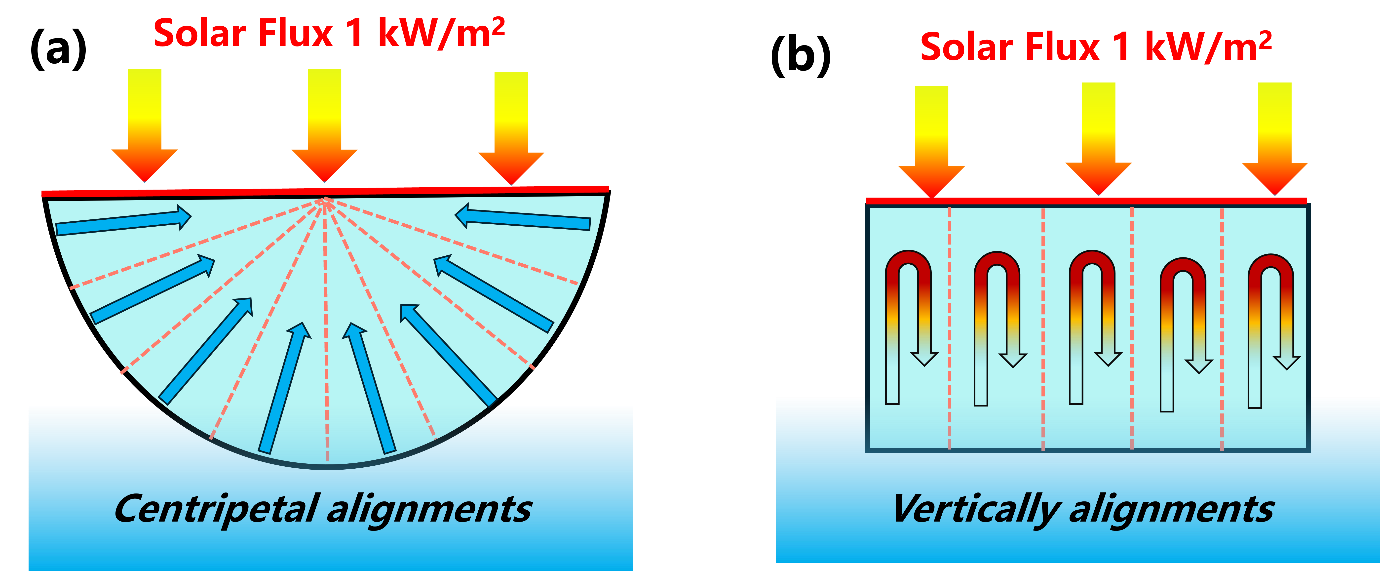


**Figure S13.** Illustration of conductive heat loss of STHE with centripetal alignments (a) and ST-V with vertical alignments (b).

**
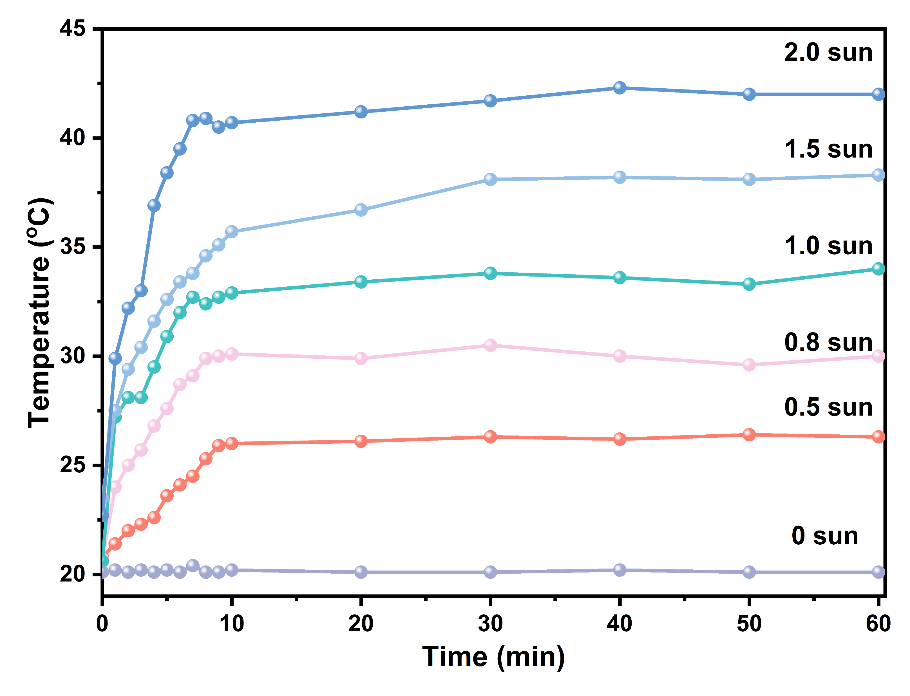
**

**Figure S14**. The corresponding surface temperature of STHE under 0 to 2.0 sun illumination.


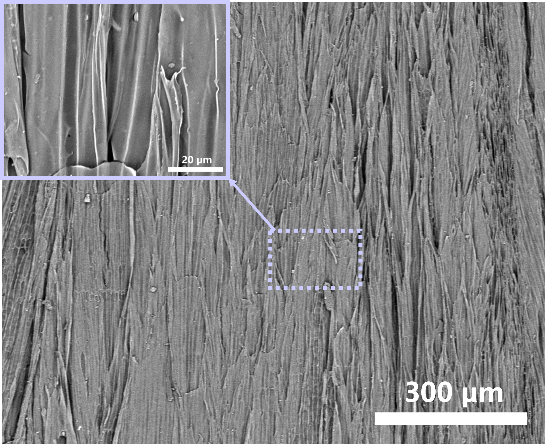


**Figure S15**. SEM images of surface of STHE after solar-driven desalination in 15.0 wt% NaCl solution under one sun illumination for 1h.


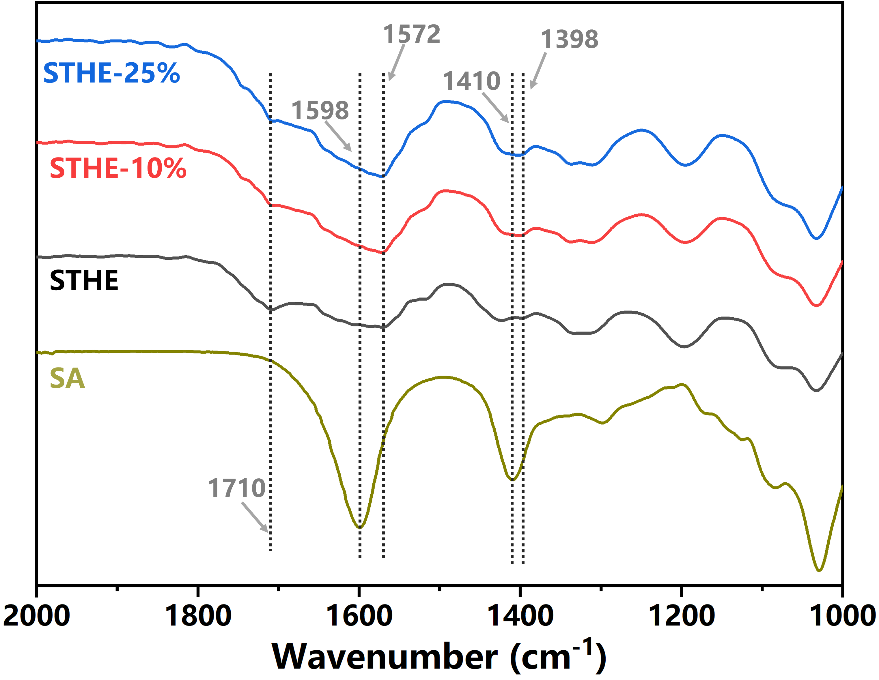


**Figure S16**. FT-IR spectra of SA, STHE, and the STHEs after dipping in 10 % and 25 % NaCl solution.

In the FT-IR spectrums, bands of pure SA appear around 1598 and 1410, corresponding to the asymmetric and symmetric –COO⁻, respectively. After SA-TA was crosslinked with Fe^3+^, the symmetric -COO⁻ peak shifted to higher wavenumbers (from 1410 to 1398 cm⁻¹), while the asymmetric -COO⁻ peak shifted to lower wavenumbers (from 1598 to1572 cm⁻¹).^[19]^ And the width of these peaks broadens considerably, and their relative intensity decreases significantly compared to pure SA, indicating the formation of strong coordination bonds between Fe^3^⁺ ions and alginate chains. When cross-linking alginate hydrogels, trivalent Fe forms stronger bonds with carboxylic acid groups than the commonly used Ca²⁺, leading to a more robust cross-linking structure.^[20]^ Due to stable crosslinked networks of SA-TA-Fe^3+^, these coordination bonds between SA-TA-Fe^3+^ remain intact without significant changes, demonstrating that no ion exchange occurs within the SA-TA Fe^3+^ networks, even after immersing STHEs in NaCl solutions (with high concentration of 10 and 25%).

**
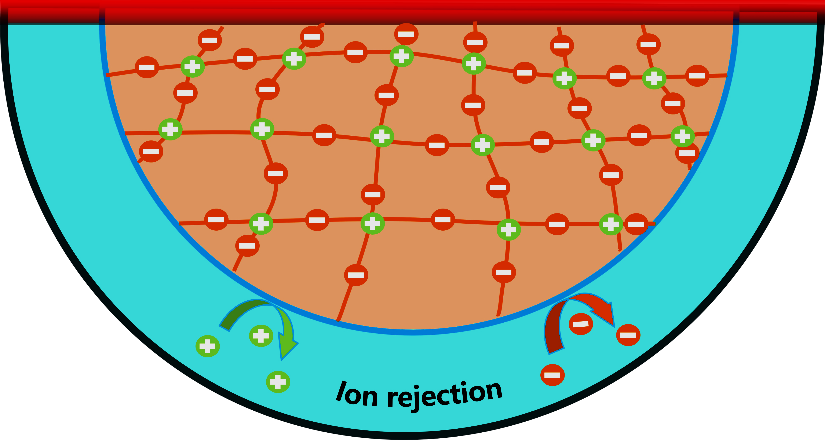
**

**Figure S17**. Ion rejection mechanism of STHE.


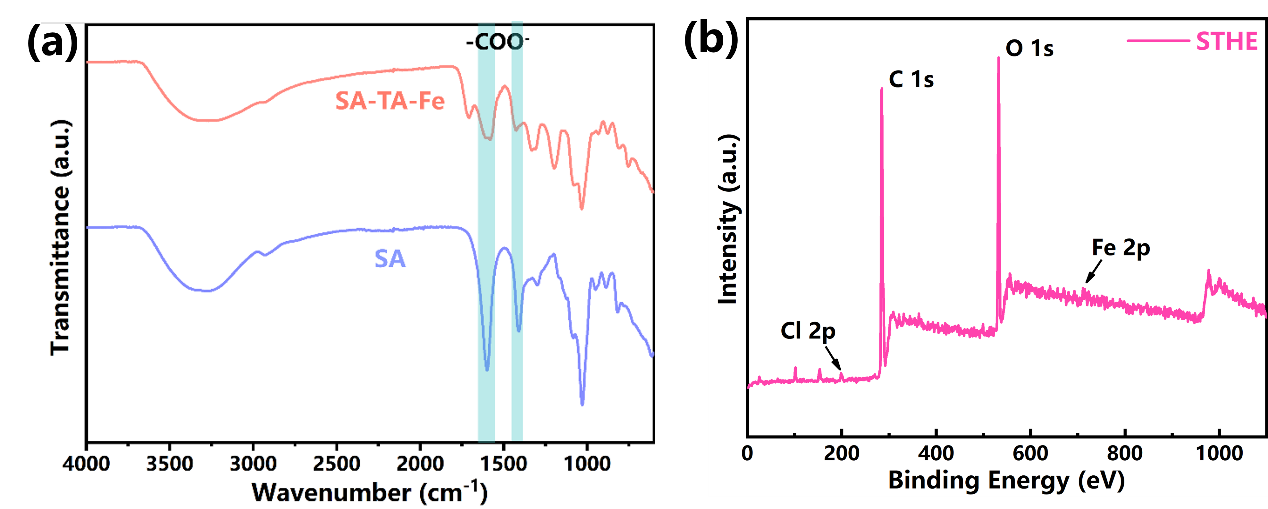


**Figure S18.** FT-IR spectra of SA and STHE (a) and XPS spectra of STHE (b).

As shown in Figure S11 (a), bands of pure SA appear around 1598 and 1410 cm^-1^, corresponding to the asymmetric and symmetric –COO⁻, respectively. After SA-TA was crosslinked with Fe^3+^, the symmetric -COO⁻ peak remains at 1398 cm^-1^ and asymmetric -COO⁻ peak at 1572 cm^-1^. This provides clear evidence for the presence and abundance of -COO⁻ groups in the crosslinked SA-TA-Fe network.

The XPS results in Figure S11 (b) show that reveals that carbon (C), oxygen (O), iron (Fe), and chlorine (Cl) are the predominant elements present. Given that the SA-TA matrix is gelatinized and chelated by ferric (III) chloride, the appearance of Fe 2p and Cl 2p peaks in the XPS spectrum of the STHE confirms the incorporation of ferric and chloride ions into the structure. This will help verify the presence of anions within the network.


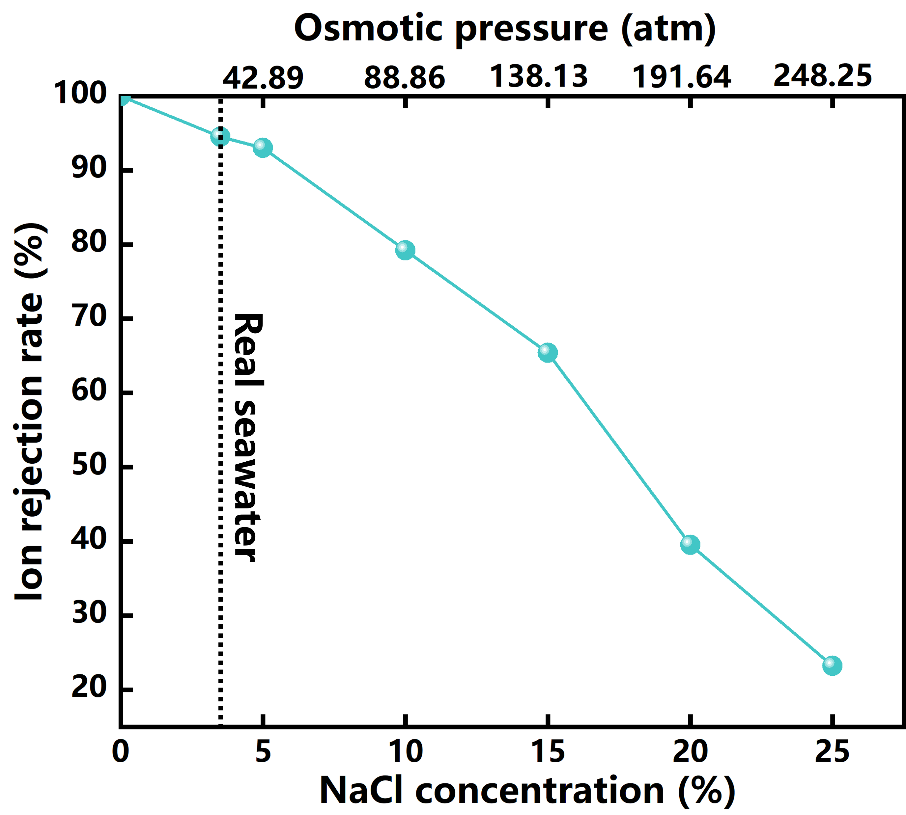


**Figure S19**. Calculated ion rejection rate of STHE in NaCl solution with different concentrations. Upper lateral axis refers to calculated osmotic pressure of NaCl solution.

Directly calculating the osmotic pressure of the hydrogel is challenging. Therefore, rather than directly determining the osmotic pressure of the STHE, its ion rejection rate can be assessed by comparing the NaCl concentration in the original solution with the concentration absorbed by the STHE at equilibrium (shown in Note S4). When the NaCl concentration is 3.5 wt%, the ion rejection rate reaches approximately 94.5%. Even as the NaCl concentration approaches saturation (25 wt%), the STHE maintains an ion rejection rate of around 23.2%. The osmotic pressure of a saturated NaCl solution can be calculated using the formula ∏=*i*CRT, where *i* is the dimensionless van’t Hoff index (2 for NaCl), R is the ideal gas constant, and T is the temperature in Kelvin, resulting in an osmotic pressure of approximately 248.25 atm (25 wt%). The STHE demonstrates a high ion rejection rate even under this condition, suggesting that its osmotic pressure might be significantly higher than that of the 25 wt % NaCl solution (~248.25 atm). This high osmotic pressure effectively prevents the entry of ions from the tested solution, contributing to its efficient desalination performance. Thus, it can be concluded that the abundant anions and cations within the SA-TA networks generate a significant osmotic pressure, which repels ions and effectively prevents salt clogging.

**
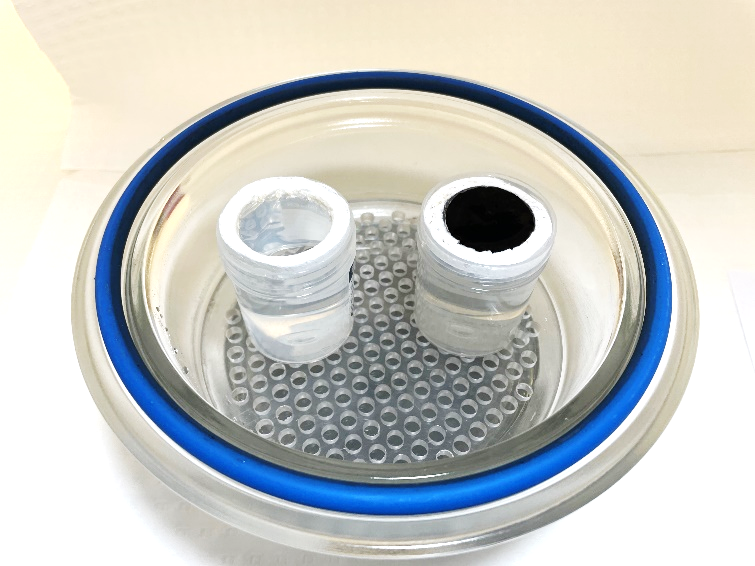

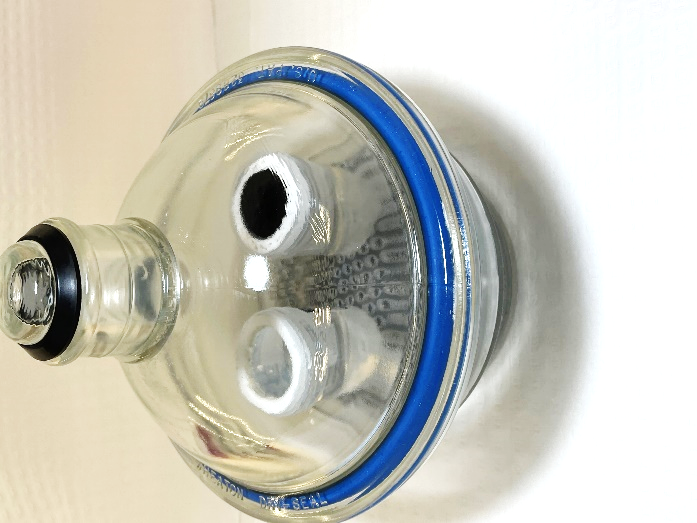
**

**Figure S20**. Schematic diagram of dark evaporation experiments of pure water and STHE in pure water.


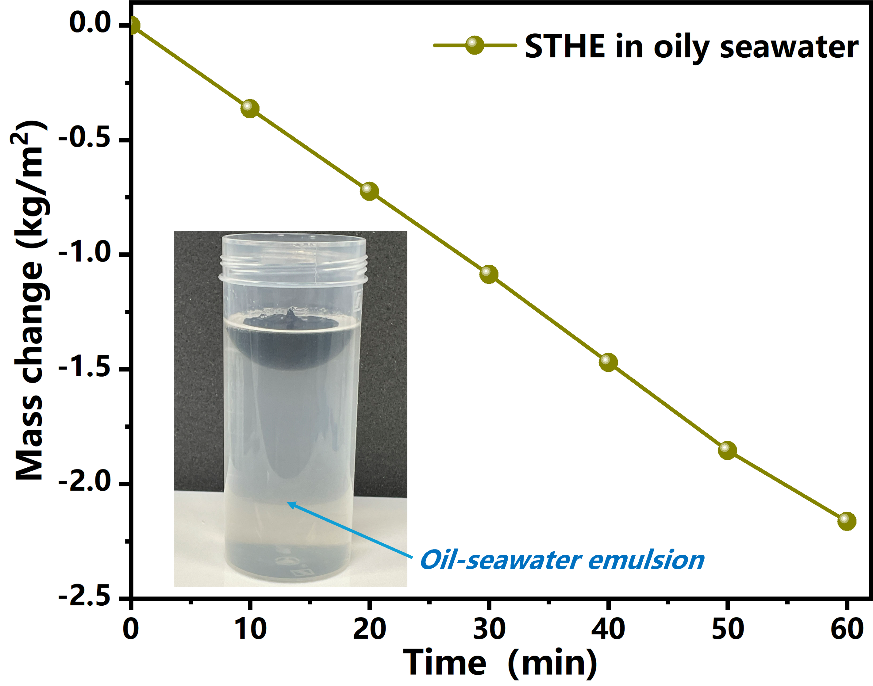


**Figure S21**. Mass change of oily seawater over time of STHE after 12-h immersion.


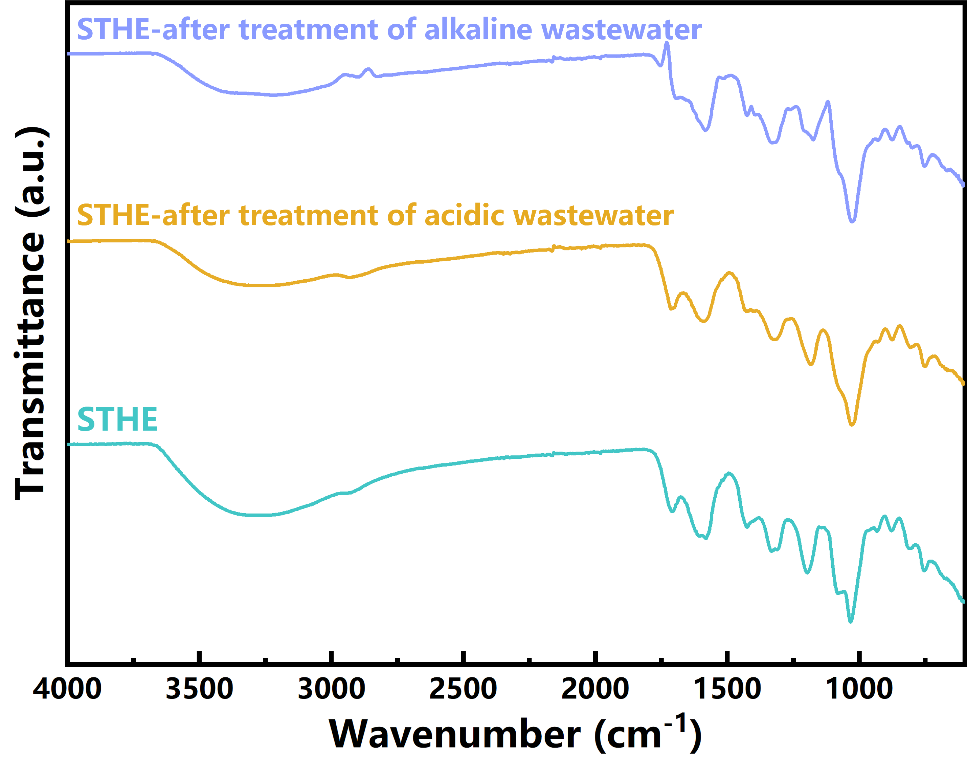


**Figure S22**. FT-IR spectra of original STHE, and the STHEs after treatment of acid and alkaline wastewater.


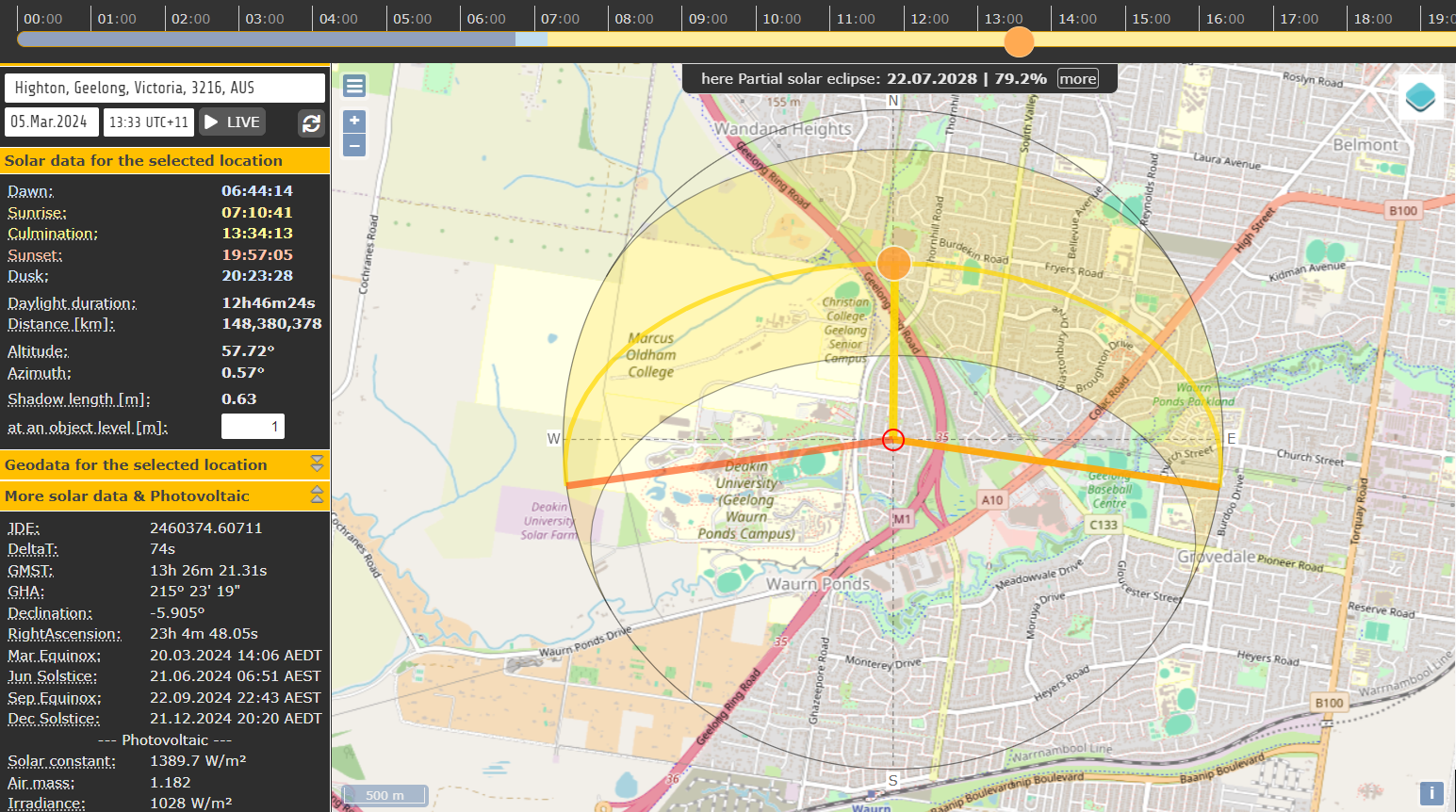


**Figure S23**. Solar data over time (Source from SunCalc.org).


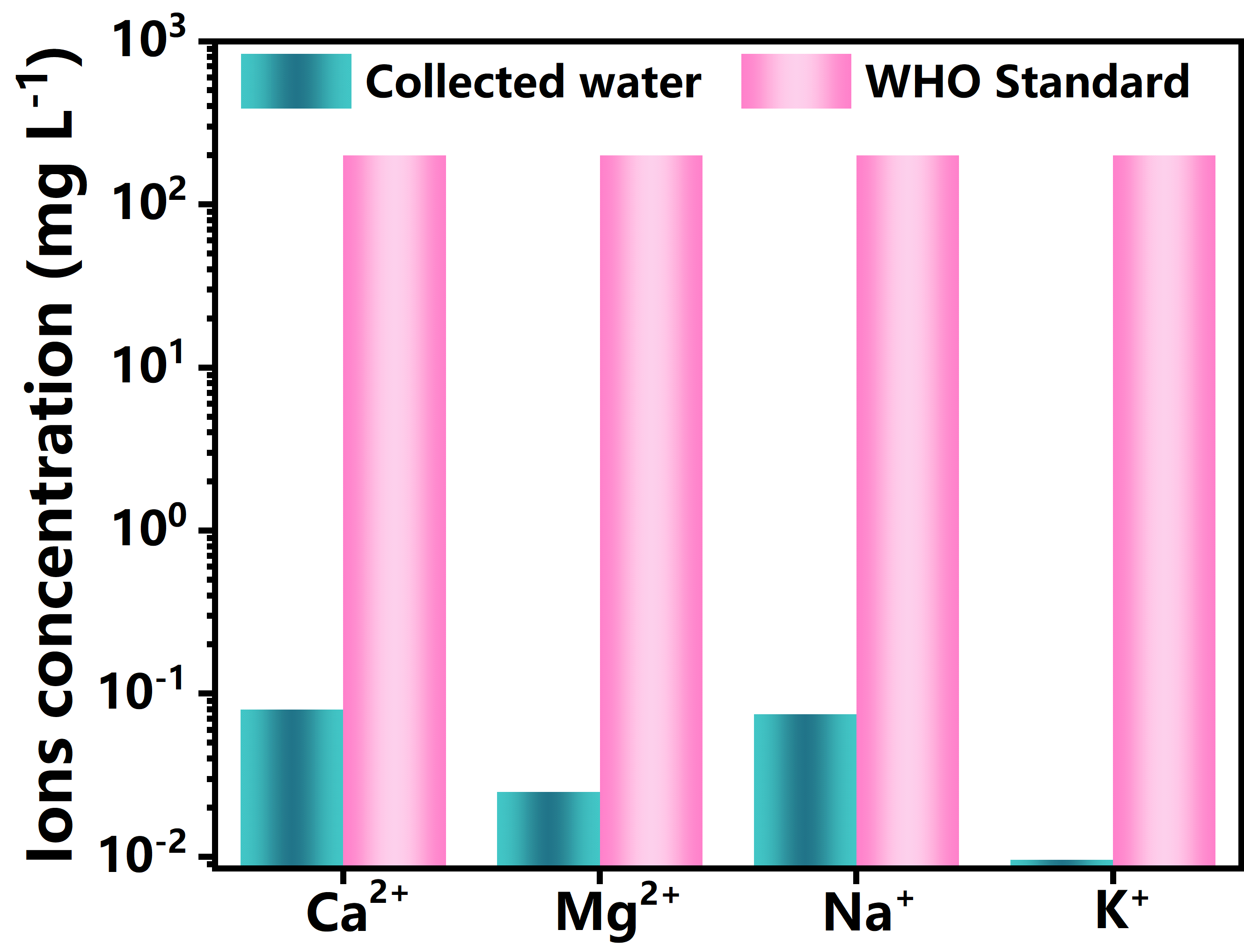


**Figure S24**. The quality of photothermal desalinated water from STHE compared with WHO standards.


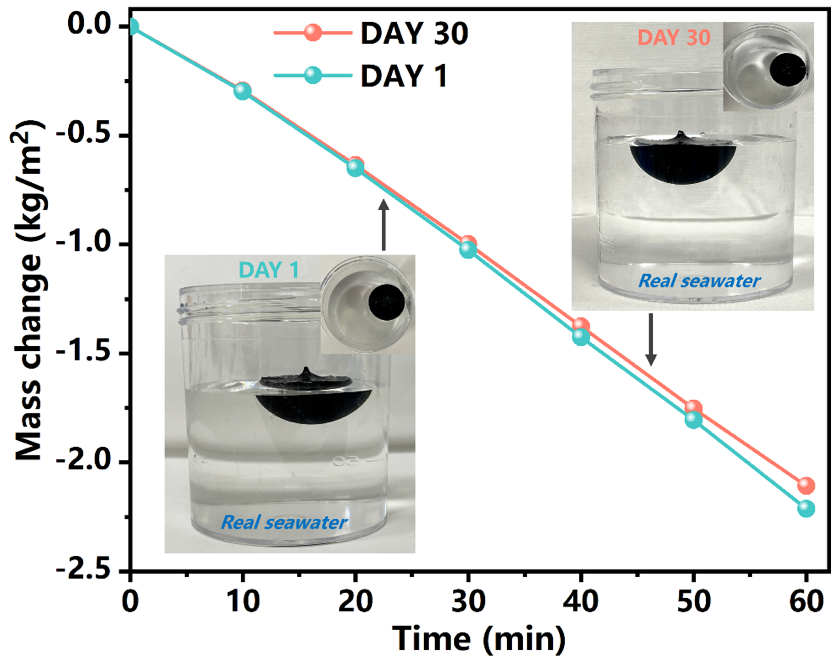


**Figure S25**. Real seawater mass change over time under one sun illumination of STHE before and after soaking it in seawater for 1 month (insets: the digital pictures of STHE soaked in real seawater at day 1 and day 30).


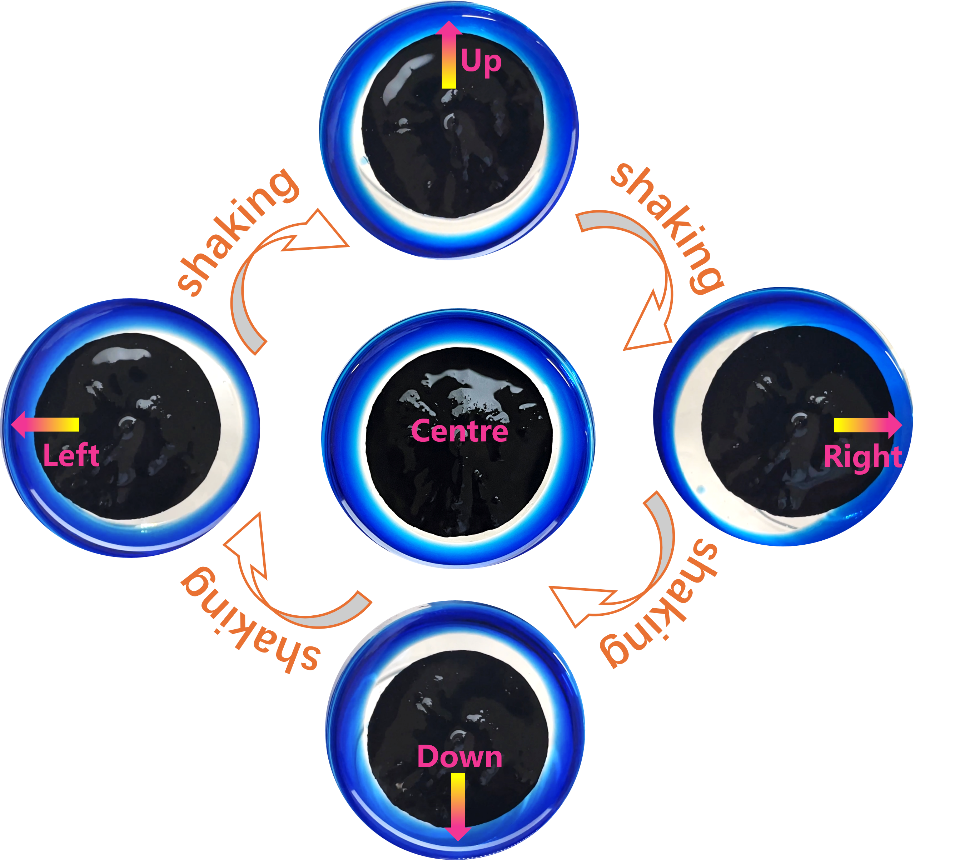


**Figure S26**. Top view of digital images the STHE floating on water surface, with blue-dyed oil floating on the water to trace the hand-induced shaking direction.

**Reference:**

[1] X. Hao, H. Yao, P. Zhang, Q. Liao, K. Zhu, J. Chang, H. Cheng, J. Yuan, L. Qu, Multifunctional solar water harvester with high transport selectivity and fouling rejection capacity. Nature Water 2023, **1** (11), 982.

[2] P. Zhu, L. Wang Microfluidics-enabled soft manufacture of materials with tailorable wettability. Chemical Reviews. 2021,**122**(7),7010.

[3] Y. Bu, X. Li, W. Lei, H. Su, H. Yang, W. Xu, J. Li, Bioinspired topological design with unidirectional water transfer for efficient atmospheric water harvesting. J. Mater. Chem. A 2023, **11** (28), 15147.

[4] Y. Kuang, C. Chen, S. He, E.M. Hitz, Y. Wang, et al. A High-Performance Self-Regenerating Solar Evaporator for Continuous Water Desalination. Adv. Mater. **31**(23), e1900498 (2019).

[5] X. Dong, Y. Si, C. Chen, B. Ding, and H. Deng. Reed Leaves Inspired Silica Nanofibrous Aerogels with Parallel-Arranged Vessels for Salt-Resistant Solar Desalination. ACS Nano **15**(7), 12256-12266 (2021).

[6] L. Song, L. Geng, Y. Tian, P. Mu, and J. Li. Robust Superhydrophilic Attapulgite-Based Aligned Aerogels for Highly Efficient and Stable Solar Steam Generation in Harsh Environments. J. Mater. Chem. A **9**(40), 23117-23126 (2021).

[7] Q. Zhang, G. Yi, Z. Fu, H. Yu, S. Chen, et al. Vertically Aligned Janus MXene-Based Aerogels for Solar Desalination with High Efficiency and Salt Resistance. ACS Nano. **13**(11), 13196-13207 (2019).

[8] X. Dong, L. Cao, Y. Si, B. Ding, and H. Deng. Cellular Structured CNTs@SiO2 Nanofibrous Aerogels with Vertically Aligned Vessels for Salt-Resistant Solar Desalination. Adv. Mater. **32**(34), e1908269 (2020).

[9] Z.Y. Wang, Y.J. Zhu, Y.Q. Chen, H.P. Yu, and Z.C. Xiong. Bioinspired Aerogel with Vertically Ordered Channels and Low Water Evaporation Enthalpy for High-Efficiency Salt- Rejecting Solar Seawater Desalination and Wastewater Purification. Small **19**(19), e2206917 (2023).

[10] J. He, Z. Zhang, C. Xiao, F. Liu, H. Sun, et al. High-Performance Salt-Rejecting and Cost-Effective Superhydrophilic Porous Monolithic Polymer Foam for Solar Steam Generation. ACS Appl. Mater. Interfaces **12**(14), 16308-16318 (2020).

[11] X. Wang, L. Zhang, D. Zheng, X. Xu, B. Bai, et al. A Polyelectrolyte Hydrogel Coated Loofah Sponge Evaporator Based on Donnan Effect for Highly Efficient Solar-Driven Desalination. Chem. Eng. J. 462, 142265 (2023).

[12] L. Li, N. He, B. Jiang, K. Yu, Q. Zhang, et al. Highly Salt-Resistant 3D Hydrogel Evaporator for Continuous Solar Desalination via Localized Crystallization. Adv. Funct. Mater. **31**(43), 2104380 (2021).

[13] Y. Bu, Y. Zhou, W. Lei, L. Ren, J. Xiao, et al. A bioinspired 3D solar evaporator with balanced water supply and evaporation for highly efficient photothermal steam generation. J. Mater. Chem. A. **10**(6), 2856-2866 (2022).

[14] M. Zou, Y. Zhang, Z. Cai, C. Li, Z. Sun, et al. 3D Printing a Biomimetic Bridge-Arch Solar Evaporator for Eliminating Salt Accumulation with Desalination and Agricultural Applications. Adv. Mater. **33**(34), e2102443 (2021).

[15] H. Jang, J. Choi, H. Lee, and S. Jeon. Corrugated Wood Fabricated Using Laser-Induced Graphitization for Salt-Resistant Solar Steam Generation. ACS Appl. Mater. Interfaces. **12**(27), 30320-30327 (2020).

[16] Z. Chen, Y. Luo, Q. Li, and X. Chen. Microgroove-Structured PDA/PEI/PPy@PI-MS Photothermal Aerogel with a Multilevel Water Transport Network for Highly Salt-Rejecting Solar-Driven Interfacial Evaporation. ACS Appl. Mater. Interfaces. **13**(34), 40531-40542 (2021).

[17] Z. Wang, Z. Zhan, Y. Li, M. Xie, H. Kong, et al. A Tree-Root Mimicked Janus Evaporator for Solar Evaporation of Saturated Saline Water. J. Mater. Chem. A. **11**(48), 26592-26601 (2023).

[18] X. Zhao, H. Zhang, K. Chan, X. Huang, Y. Yang, et al. Tree-Inspired Structurally Graded Aerogel with Synergistic Water, Salt, and Thermal Transport for High-Salinity Solar-Powered Evaporation. Nano-Micro Lett. **16**, 222(2024).

[19] Zhang, L., Zhang, L., Chai, L., Xue, P., Hao, W., & Zheng, H. (2014). A coordinatively cross-linked polymeric network as a functional binder for high-performance silicon submicro-particle anodes in lithium-ion batteries. J. Mater. Chem. A, ***2***(44), 19036-19045.

[20] Zhao, Z., Wang, C., Wei, D., Hu, Q., Tan, P., Wang, F., & Zhang, J. (2024). Tortuosity Engineering of Water Channels to Customized Water Supply for Enhancing Hydrogel Solar Evaporation. Small, 2402482.
